# Novel Psychoactive Substances: The Razor’s Edge between Therapeutical Potential and Psychoactive Recreational Misuse

**DOI:** 10.3390/medicines9030019

**Published:** 2022-03-01

**Authors:** Beatriz Correia, Joana Fernandes, Maria João Botica, Carla Ferreira, Alexandre Quintas

**Affiliations:** 1 Laboratório de Ciências Forenses e Psicológicas Egas Moniz, Campus Universitário—Quinta da Granja, Monte de Caparica, 2825-084 Caparica, Portugal; beatriz.marques.correia@gmail.com (B.C.); wwwjoana2010@hotmail.com (J.F.); cferreira@egasmoniz.edu.pt (C.F.); 2 Instituto Português de Oncologia de Lisboa Francisco Gentil (IPO), Alameda Prof. Hernâni Monteiro, 4200-319 Porto, Portugal; mjt.botica@gmail.com; 3 Molecular Pathology and Forensic Biochemistry Laboratory, Centro de Investigação Interdisciplinar Egas Moniz, 2825-084 Caparica, Portugal; 4 Faculty of Medicine of Porto University, Rua Professor Lima Basto, 1099-023 Lisboa, Portugal

**Keywords:** NPS, therapeutic, toxicology, synthetic cathinones, synthetic cannabinoids

## Abstract

Background: Novel psychoactive substances (NPS) are compounds of natural and synthetic origin, similar to traditional drugs of abuse. NPS are involved in a contemporary trend whose origin lies in a thinner balance between legitimate therapeutic drug research and legislative control. The contemporary NPS trend resulted from the replacement of MDMA by synthetic cathinones in ‘ecstasy’ during the 2000s. The most common NPS are synthetic cannabinoids and synthetic cathinones. Interestingly, during the last 50 years, these two classes of NPS have been the object of scientific research for a set of health conditions. Methods: Searches were conducted in the online database PubMed using boolean equations. Results: Synthetic cannabinoids displayed protective and therapeutic effects for inflammatory, neurodegenerative and oncologic pathologies, activating the immune system and reducing inflammation. Synthetic cathinones act similarly to amphetamine-type stimulants and can be used for depression and chronic fatigue. Conclusions: Despite the scientific advances in this field of research, pharmacological application of NPS is being jeopardized by fatalities associated with their recreational use. This review addresses the scientific achievements of these two classes of NPS and the toxicological data, ending with a reflection on Illicit and NPS control frames.

## 1. Introduction

Over the past 15 years, the market for psychotropic substances has been flooded with a vast and heterogeneous set of substances active over the central nervous system (CNS), termed novel psychoactive substances (NPS) [1]. According to the United Nations Office on Drugs and Crime (UNODC), NPS encompass substances not controlled by the 1961 Single Convention on Narcotic Drugs, the 1971 Convention on Psychotropic Substances and the 1988 Convention against Illicit Traffic in Narcotic Drugs and Psychotropic Substances. These compounds, of natural and synthetic origin, mimic the effects of traditional drugs of abuse, posing a risk to public health due to the lack of pharmacological knowledge [2]. These compounds are distinguished from the classical drugs, such as amphetamine, cocaine, heroin, and cannabis, due to their poor or absent medicinal historical use [3]. Thus, the term ‘novel’ on NPS does not necessarily refer to original molecules but to substances that have recently become available on the recreational market [4]. In Europe, at least one new substance is detected weekly on the drug market [5]. 

Interestingly, NPS owe their origin to a conflict between socioeconomic changes, legislation revision and legitimate drug research with medical purposes on opiates. In 1898, the Bayer Company started the production of heroin by deacetylation of morphine and was mistakenly pronounced to be free from abuse liability. Throughout the 1910s and 1920s, the U.S. placed restrictions on opiates, requiring formal prescriptions to be sold, and simultaneously banned heroin [6,7]. In the 1930s, amphetamine, which was first synthesized in 1887 by Lazar Edeleano [8], started to be used due to its pharmacological effects on attention and cognition, emotions and appetite. It was spread as an antidepressant during the next decade, gaining success as a medication to lose weight [9,10,11]. The nonmedical use of amphetamines and their addiction potential led to strict control by the United Nations (UN) Convention of 1971 [12]. Further, 3,4-methylenedioxymethamphetamine (MDMA), a derivative of amphetamine also known as ‘ecstasy’ or ‘molly’, was first synthesized in 1912 and patented by Merck in 1914 [13]. This substance gained popularity in psychiatric practice during the 1970s and 1980s due to its empathogenic effect, making patients more open to psychotherapy [13,14,15,16]. MDMA was used as a recreational drug associated with electronic dance music events and all-night dance parties during the same period, termed raves. ‘Ecstasy’ reached its peak of usage during the 1980s. MDMA became an internationally controlled substance in 1986 [17]. The measures against MDMA and its precursors led to a worldwide shortage of this substance in the mid-2000s. The continuing demand for ecstasy led to alternatives to MDMA, including ‘ecstasy’ pills, which mimicked its effects. The substances found as substitutes for MDMA included synthetic cathinones, such as mephedrone, among others, marking the current boom of NPS [18].

The introduction of synthetic cathinones as recreational substances, in ‘ecstasy’ and ‘bath salts’ on the market [19], was accompanied by other classes of compounds mimicking the effects of opiates, benzodiazepines, dissociative, hallucinogens and cannabinoids [20,21]. Compared to traditional drugs of abuse, NPS are cheaper, easier to buy, and their transitory unregulated status makes them very attractive, especially among young people [22]. The compound JWH-018 was among the first NPS found in the streets [23]. The synthetic cannabinoid, when added to smokeable plant material, is branded as ‘spice’ [24,25]. Synthetic cannabinoids research began after the UN acts to control narcotics and psychotropic substances. Due to these restrictions in the early 1980s, Pfizer© and Lilly© developed several synthetic cannabinoids mimicking the effects of Δ^9^-tetrahydrocannabinol (Δ^9^-THC), aiming to understand the endocannabinoid system [26]. Subsequently, many other cannabinoid-like molecules have been synthesized with the purpose to develop novel therapeutic strategies against Alzheimer’s and Parkinson’s diseases, arthritis, colitis and several oncological conditions [27,28,29,30,31,32,33,34]. Despite the past and present research efforts, the Food and Drug Administration (FDA) just approved Dronabinol, Nabilone and Rimonabant, and this last one was already removed from the market [35].

According to the European Monitoring Centre for Drug and Drug Addiction (EMCDDA), NPS seizures are dominated by synthetic cannabinoids and synthetic cathinones, up to 60% of NPS confiscations in Europe in 2019 [36]. By the end of 2020, around 209 synthetic cannabinoids and 156 synthetic cathinones had been identified in Europe and reported by EMCDDA [36]. Synthetic cannabinoids are typically found in herbal blends or, less typically, in the form of tablets, capsules and powders [37]. More recently, liquid products have emerged for e-cigarettes [38]. Otherwise, synthetic cathinones are typically found in powder [39]. The accurate components of the selling package are unknown since the advertising and packaging of these products are often misleading, with evidence showing that NPS is causing global short- and long-term health problems [21]. The present review reflects on the scientific achievements of using NPS in medical research, focusing on the mechanisms of diseases. It also brings forward the thin line separating legitimate medical research from substance abuse in light of the contemporaneous trend of NPS, allowing to better understand the origins of such trends.

## 2. Materials and Methods

Searches were conducted in the online database PubMed, and an advanced search was performed using the following boolean equations: (i) “synthetic cannabinoids” AND “therapeutics” with a cut-off filter to select just the papers from the last 20 years; (ii) “synthetic cathinones” AND “therapeutics”; (iii) “synthetic cannabinoids” AND “toxicity”; (iv) “synthetic cathinones” AND “toxicity”. The search was limited to English-language peer-reviewed journal publications. In the exclusion criteria, papers that did not focus on synthetic cannabinoids or cathinones or had no relationship between them and toxicity/therapeutic were excluded. Different sources were identified by following up internal citations and references within the documents retrieved in the initial search.

## 3. Most-Consumed NPS

### 3.1. Synthetic Cathinones

Synthetic cathinones are derived from cathinone, which is the principal active ingredient in the leaves of the khat plant (*Catha edulis*). These substances act similarly to amphetamine-type stimulants, promoting euphoria, mood elevation, sense of wellbeing, energy, wakefulness, fatigue decrease, focus and alertness increase [40]. Ephedrone (methcathinone) and mephedrone (4-methyl-methcathinone) were the first cathinone derivatives to be produced [41]. Notwithstanding the medical research for the therapeutic use of synthetic cathinones, just bupropion and amphepramone (N,N-diethylcathinone) are still used as medicine as antidepressants and appetite suppressants, respectively. Synthetic cathinones appeared in drug markets in the mid-2000s as alternatives to MDMA or ‘ecstasy’ [42].

Synthetic cathinones are commonly known as bath salts and are available in online drug stores under various brand names, including Bliss, Cloud Nine, Lunar Wave and Vanilla Sky [43,44]. Generally, considering the mechanism of action and similarity to classic drugs, synthetic cathinones are divided into three subgroups [45] The first subgroup consists of cathinones with similar effects to cocaine and ecstasy (e.g., mephedrone, methylone, ethylone and butylone). Like cocaine, these compounds inhibit the reuptake of monoamines, prominently of dopamine (DA), and also mimic the action of MDMA by stimulating the release of serotonin (5-HT). The second subgroup consists of methamphetamine-like cathinones (e.g., 4-methylcathinone and flephedrone (4-FMC)), which, like amphetamine and methamphetamine, inhibit the reuptake of DA and noradrenaline (NA) and stimulate the release of DA. Finally, the third one is the pyrovalerone type of cathinones (e.g., 3,4-methylenedioxypyrovalerone (MDPV), α-pyrrolidinopentiophenone (α-PVP or ‘flakka’) and α-pyrrolidinoheptaphenone (PV8)), which strongly inhibit DA and NA reuptake, not affecting the release of monoamines [45,46].

Due to the continuous search for new, legal, less expensive and more powerful effects, the synthesis of novel cathinone derivatives became a fruitful industry, leading to the fast emergence of new alternative substances every year. Moreover, the decrease in availability and purity of the more typical abusable drugs is advantageous for the illicit drug market to introduce synthetic cathinones [47]. As earlier synthetic cathinones were banned, a group of products named ‘energy’ (NRG), advertised as naphthyl cathinone analogues, emerged in 2010 [41]. During the same period, another type of synthetic cathinones started showing up, first 3,4-dimethylmethcathinone (3,4-DMMC) in 2010, then pentedrone (α-methylaminovalerophenone) and, finally, α-PVP in 2013 [5,48]. Moreover, the drug market was flooded by other psychoactive cathinone derivatives to circumvent the law. Para substituted cathinone derivatives, such as 4-FMC, brephedrone (4-BMC) and methedrone (PMMC), showed up on the illicit drug market and increased recreational drug misuse. Recently, clephedrone (4-chloromethcathinone, 4-CMC) and clophedrone (3-chloromethcathinone, 3-CMC), chloride substituted derivatives of cathinone, were offered on the internet for the first time [49]. However, 4-CMC is already controlled under the 1971 UN Convention on Psychotropic Substances because of the public health and social risks that it poses [50]. However, there are also synthetic cathinones used as medicine, such as bupropion and amphepramone.

### 3.2. Synthetic Cannabinoids

Synthetic cannabinoids are a group of NPS with similar properties to Δ^9^-THC and other cannabinoids naturally found in the *Cannabis sativa L.* and *Cannabis indica* plants. The effects promoted by these substances are similar to the ones caused by the phytocannabinoids they mimic [51]. However, such as with cannabis, the effect depends on the person exposed to it. Consequently, while some users may feel euphoric, relaxed and talkative, others may feel ill or paranoid [51,52,53,54]. Synthetic cannabinoids are often more potent than phytocannabinoids, so their use is frequently associated with unpleasant experiences and harmful effects [55,56]. Cannabinoid receptor agonists have been developed for therapeutic purposes after the UN Conventions to control narcotics and psychotropic substances [57]. However, most of these substances were not approved by medicine regulatory agencies. The only synthetic cannabinoids used medically are nabilone and dronabinol [58,59,60]. However, despite its synthetic origin, the dronabinol pharmacological active ingredient is Δ^9^-THC [59]. In the early 2000s, these substances were found as an herbal blend in the street drug recreational market [42].

According to UNODC, synthetic cannabinoids can be divided into seven classes based on their structural differences, lipophilicity and binding capacity to cannabinoid receptors [61]. These are the classic cannabinoids, the non-classical cannabinoids, the hybrid cannabinoids, the aminoalkylindols, the aminoalkylindazoles, the eicosanoids and others. Synthetic cannabinoids can also be grouped into different series according to the entity responsible for their introduction and synthesis, including the series JWH (John William Huffman), aminoalkylindols and AM (Alexandros Makriyannis) and HU (Hebrew University) classic cannabinoids. The HU and AM series, such as the HU-210, AM-906 and AM-411, and the O-1184 are designated classical cannabinoids since they derive from the structure of phytocannabinoids [62]. Despite the chemical heterogeneity among the several classes of synthetic cannabinoids, they act by biding to cannabinoid receptors [63]. There are two known cannabinoid receptors subtypes, cannabinoid receptor 1 (CB1) and cannabinoid receptor 2 (CB2) [64].

Although the research on synthetic cannabinoids aims to develop new compounds for therapeutic purposes, this group of substances has quickly become popular in the recreational market under the designation of ‘spice’ [57]. JWH-018 was the first synthetic cannabinoid present in spice reported to the EMCDDA in December 2008 [23,65,66,67].

## 4. Therapeutic Potential of Synthetic Cathinones and Synthetic Cannabinoids

### 4.1. Synthetic Cathinones

Synthetic cathinones-related effects bank on two primary mechanisms: monoamine uptake blockade resulting from transporter inhibition and increased monoamine release. In addition, the effects can be derived from the two mechanisms combined [68]. Regardless of the molecular mechanism involved, all synthetic cathinones increase extracellular monoamine concentrations in the brain, enhancing cell-to-cell monoamine signalling, and are potent inhibitors of NA transporter (NET) [69]. However, they differ in their inhibition profiles on DA transporter (DAT) and 5-HT transporter (SERT) and in their ability to release monoamines, which possibly explains clinical differences reported in their effects and toxicities [68,70,71,72]. Cathinone highly inhibits DAT but is a less potent SERT inhibitor [70]. MDPV is one of the first recreational synthetic cathinones and acts as a potent, selective monoamine uptake blocker, with high affinity for DAT and NET and weak for SERT, but has less impact on monoamine release than cocaine [71]. Still, comparing the uptake blocking capacity of both, MDPV is 10 and 50 times more potent as an uptake blocker than DAT and NET, respectively [68]. On the other hand, mephedrone and methylone act as nonselective monoamine uptake inhibitors [71] like cocaine and increase serotonin release like MDMA [72].

The observation of synthetic cathinones’ effects on CNS led to the study of their pharmacology and, eventually, their medical application to treat depression, chronic fatigue and obesity [73,74]. Otherwise, synthetic cathinones have also been used by undiagnosed attention-deficit/hyperactivity disorder (ADHD) adolescents, who self-medicate with synthetic cathinones [75].

#### 4.1.1. Depression

Depression is a common illness that affects an estimated 3.8% of the world population (approximately 280 million people) [76]. Depression was ranked by the World Health Organization (WHO) as the single most significant contributor to global disability [77]. Common symptoms are sadness, irritability, emptiness or loss of interest in activities [78]. Depression is often attributed to a defective serotonergic function, but an ineffective compensatory response to abnormally high serotonergic function can also be responsible [79]. Especially when recurrent and with moderate or severe intensity, depression may become a serious health condition and, at its worst, may lead to suicide [77,80].

Synthetic cathinones, namely methcathinone and bupropion, might be used in the treatment of depression. Methcathinone was used as an antidepressant in the 1930s and 1940s, and bupropion is still prescribed for the treatment of depression and smoking cessation by the FDA [73,81].

#### 4.1.2. Chronic Fatigue

Lethargy and chronic fatigue are complex multisystemic diseases characterized by severe fatigue, cognitive dysfunction, sleep difficulties and autonomic dysfunction [82]. In the 1970s, the synthetic cathinone pyrovalerone started to be used to treat lethargy and chronic fatigue [74]. This medicine is still an approved medication by the FDA. However, it is a Schedule V controlled substance and is rarely prescribed [69].

#### 4.1.3. Obesity

Obesity is a worldwide issue that has nearly tripled since 1975. In 2016, more than 1.9 billion adults were overweight, and 39 million children under the age of 5 were overweight or obese in 2020 [83]. Obesity is a complex, multifactorial preventable disease primarily associated with excess adiposity, or body fatness [84]. This condition considerably increases the risk of chronic disease morbidity, disabilities, depression, type 2 diabetes, cardiovascular disease, certain types of oncologic pathologies and mortality [85]. If the secular trends continue, by 2030, an estimated 38% of the world’s adult population will be overweight and another 20% will be obese [86].

In the past, two synthetic cathinones were used as appetite suppressants, namely amphepramone and metamphepramone (N,N-dimethylcathinone or dimethylpropion) [87,88,89]. Currently, only amphepramone is still in use.

#### 4.1.4. Attention-Deficit/Hyperactivity Disorder

ADHD is among the most common neurobehavioral disorders in children. The clinical significance of the signs and symptoms of the disorder has been recognized for over two centuries [90]. It carries a high rate of comorbid psychiatric difficulties, such as oppositional defiant disorder (ODD), conduct disorder, mood and anxiety disorders, and also the consumption of substances of abuse. The societal costs of untreated ADHD are significant, including academic and occupational underachievement, delinquency, motor vehicle safety, difficulties with personal relationships and excessive talking with impaired listening comprehension [90]. Scientists have not yet identified the specific causes of ADHD. Still, there is evidence that genetics contribute to the disorder, but factors such as being born prematurely, brain injury or the mother smoking, using alcohol or having extreme stress during pregnancy can also be involved [91]. The main features of the ADHD diagnosis are (1) the presence of inappropriate levels of hyperactive–impulsive and/or inattentive symptoms for at least 6 months; (2) symptoms occurring in different settings, and that cause impairments in living; (3) some of the symptoms first occurring in early to mid-childhood years of the person and (4) that no other disorder better explains the symptoms [91]. The clinical presentation of ADHD can be described as primarily inattentive, primarily hyperactive–impulsive, or combined, depending on the nature of the symptoms. Studies indicate that distraction is more powerfully associated with academic underachievement, low self-esteem, negative occupational outcomes and lower overall adaptive functioning [90]. Data reporting to 2016 described that, of the children with current ADHD, almost two-thirds were taking medication and around half of them had received behavioural treatment for ADHD in the past year [92].

Phenethylamines are a class of stimulants prescribed in patients with ADHD, namely methylphenidate. Methylphenidate and other ADHD pharmacotherapies influence the nucleus acumbens of adolescents with ADHD in the same way as cocaine. Hence, cocaine dependence in adolescents with ADHD might answer to therapeutic interventions that substitute cocaine with psychostimulants, such as MDPV [75]. Synthetic cathinones are substances with chemical structures related to phenethylamines, promoting similar effects. Nowadays, the use or potential use of cathinones to treat this disease is still a matter of debate, being hindered by the harmful secondary effects to users. However, undiagnosed ADHD adolescents often use bath salts to self-medicate, aiming to contain ADHD symptoms [93]. MDPV comes close to the effect of methylphenidate at low doses, and its self-administration can induce psychoactive effects that help alleviate ADHD symptoms, so adolescents might continue to experience enhanced concentration and overall performance [72]. It is important to note that bath salts can be found worldwide at a low cost in internet shops.

There is still a lack of knowledge around synthetic cathinones’ therapeutic potential, with a great deal of room left for new studies to develop.

### 4.2. Synthetic Cannabinoids

#### 4.2.1. Inflammatory Pathologies

Inflammation is an immune system’s response to harmful stimuli, such as pathogens, toxic compounds and damaged cells, among others, crucial for initiating the healing process [94]. However, when the molecular and cellular events underlying acute inflammation become uncontrolled, the process may become chronic, developing chronic inflammatory diseases. Such is the case of arthritis and colitis. Several studies have shown that cannabinoids downregulate cytokine and chemokine production, suppressing inflammatory responses. Thus, synthetic cannabinoids are being investigated as novel therapeutics approaches to such conditions [29,95,96,97].

##### Arthritis

Rheumatoid arthritis is an inflammatory disease characterized by persistent synovitis, painful systemic inflammation and autoantibodies, which lead to joint damage and disability [98,99]. This condition results from an overactive immune system that leads to excessive and unregulated inflammation [99,100]. As a result of this event, the tissue invasion by immune cells and their effectors, such as pro-inflammatory cytokines, is prolonged, damaging the affected areas and provoking the typical symptoms of inflammation, namely pain, rubor, warmth and swelling [100]. The pathophysiology of arthritis is related to a series of catabolic events that lead to degradation and consequent loss of articular cartilage and resorption of subchondral bone [101,102]. The pathway that leads to these events seems to involve a complex network of signalling agents, including high levels of pro-inflammatory cytokines (e.g., IL-1, IFN-γ, TNFα, IL-17) and lower levels of the anti-inflammatory factor interleukin IL-10 [103,104,105,106]. Additionally, inflammatory arthritis is associated with increased production of nitric oxide (NO) due to activation of the nitric oxide synthase (iNOS) pathway [107]. Several cell types present within the joint, including chondrocytes, can be induced by pro-inflammatory cytokines to produce NO [106]. NO production in the early stages of arthritis may cause apoptosis in chondrocytes, contributing to cartilage degradation [108,109]. Additionally, there is evidence that NO synthesis reduces proteoglycan and type II collagen synthesis, both components of the cartilage extracellular matrix [108,110].

Considering that the endocannabinoid system plays an essential role in several processes, including inflammation and immune system modulation [111], which are implicated in inflammatory arthritis pathogenesis, synthetic cannabinoids are an object of medical research as a potential treatment for this condition. In 2005, Mbvundula et al. demonstrated that R-(+)-WIN-55,212, a nonselective cannabinoid receptor agonist, reduced NO production in chondrocytes, suggesting that some cannabinoids may prevent cartilage resorption through inhibiting cytokine-induced NO production by chondrocytes and by inhibiting proteoglycan degradation [32]. A similar effect was observed with the CB agonist CP-55,940 [95], which was also proven to stimulate osteoclast formation in vitro [96]. Moreover, Gui and collaborators have shown a reduction in osteoclast formation in osteoblast-bone marrow in the presence of the synthetic cannabinoid HU-308. The mechanism seems to involve the decrease in the levels of IL-6 and TNFα through the CB2 receptor [97]. Although targeting the cannabinoid system seems to be a promising therapeutic approach, cannabis-based drugs interact with receptors other than CB receptors, having unexpected outcomes in clinical studies compared to preclinical trials.

##### Colitis

Ulcerative colitis is a chronic, idiopathic inflammatory bowel disease characterized by relapsing and remitting mucosal inflammation [112]. This inflammation starts in the rectum, extends to the colon’s proximal segments and results in diffuse friability and superficial erosions on the colonic wall and consequent bleeding [113]. The most common symptoms are blood in the stool and diarrhoea. In severe disease, the symptoms referred to can be accompanied by incontinence, increased frequency of bowel movements, abdominal discomfort, fever and others [113,114]. Both genetic and environmental factors have an essential role in ulcerative colitis development. Family history of inflammatory bowel disease has been reported as a relevant risk factor in this disease [115,116]. Moreover, factors such as drug use, changes in the gut microbiota composition and impaired mucosal immunity seem relevant in ulcerative colitis aetiology [113,117,118].

The pathophysiology of ulcerative colitis is complex since it involves impairment in the epithelial barrier, immune response, leukocyte recruitment and microflora of the colon [113,119,120]. Despite the complexity of the disease, here, just the influence of abnormal immune response and leukocyte recruitment in ulcerative colitis development is addressed. In physiological conditions, the single-layered intestinal epithelium behaves like a physical and immunological barrier that prevents direct contact between luminal microbiota and intestinal mucosa [121]. However, if the intestinal epithelium is injured, an immune response is initiated, leading to neutrophil recruitment. These neutrophils recognize, phagocytise and kill pathogenic agents and promote the production of cytokines and other pro-inflammatory factors that also regulate inflammation and immune system response [121,122,123,124,125]. Neutrophil accumulation in intestinal tissue can promote significant tissue damage when not properly eliminated [121].

In 2014, Fichna et al. demonstrated that AM-841, a preferential CB1 receptor agonist, reduces inflammation in the colon of mice with induced colitis, attenuates colitis and inhibits ulceration [29]. This effect is due to a decrease in immunocytes infiltration of the colonic tissue, improving the mucosal and muscle architecture and inhibiting its ulceration. More specifically, AM-841 inhibits fMLP-stimulated neutrophil migration, an essential feature of the anti-inflammatory action of this synthetic cannabinoid [29]. Indeed, fMLP is a chemical compound that attracts neutrophils [126]. It is worth mentioning that the AM-841 anti-inflammatory effects occur when this synthetic cannabinoid is administered prior to colitis induction, revealing its protective properties [29].

To understand AM-841 action on the cannabinoid receptors, Fichna and collaborators used mice with cannabinoid receptors CB1 and CB2 and without one or both receptors [29]. This experiment revealed that AM-841 did not attenuate colitis in mice in the absence of one or both cannabinoid receptors. As such, despite its preference for the CB1 receptor, the data suggest that just AM-841 action on both cannabinoid receptors alleviates colitis in mice [29].

Concluding, AM-841 displayed protective and therapeutic effects on colitis in mice through its anti-inflammatory action, mediated through the CB1 and CB2 receptors [29].

#### 4.2.2. Neurodegenerative Pathologies

Neurodegenerative diseases are progressive, incapacitating conditions involving the function loss of nerve cells in the brain or peripheral nervous system, affecting millions worldwide. The hallmark of these pathologies is the accumulation of misfolded and aggregated proteins associated with neuroinflammation, infection, mitochondrial dysfunction and excitotoxicity. Phytocannabinoids and synthetic cannabinoids appear to be neuroprotective either by binding to the CB1 or CB2 receptors [127] and are used worldwide by patients with neurodegenerative diseases. Therefore, cannabinoid receptor agonists are a research field for therapeutic purposes. Notwithstanding, most of these substances were not approved by medicine regulatory agencies. Thereby, the potential therapeutic of synthetic cannabinoids is explored, focusing on Parkinson’s disease (PD), Alzheimer’s disease (AD) and neurocognitive disorders associated with HIV-1 [24,28,33,34,128].

##### Parkinson’s’ Disease

PD is a chronic, progressive neurodegenerative disease characterized by the loss of dopaminergic neurons in the substancia nigra pars compacta, a midbrain dopaminergic nucleus [129,130]. This pathology is the second most common neurodegenerative disorder worldwide, and its prevalence has been rising in the last three decades [131]. Its onset occurs mainly in later life, giving rise to resting generalized tremors, bradykinesia and rigidity [130,132]. Besides these symptoms, loss of smell, sleep dysfunction, mood disorders, constipation, excessive salivation and postural instability in the latter phase of the disease can be described [129,133]. The aetiology of Parkinson’s disease is driven by a complex interplay between genetic and environmental factors [134,135].

PD is an α-synucleinopathy since it involves the abnormal accumulation of α-synuclein protein in the neuronal tissue [132]. This abnormal accumulation culminates in the generation of Lewy bodies, which leads to neuronal death in dopaminergic and non-dopaminergic brain areas. The loss of dopaminergic neurons leads to motor and non-motor symptoms that characterize PD [133,136]. Besides α-synuclein aggregation, other key molecular events have been associated with PD, such as mitochondrial dysfunction and oxidative stress, which lead to radical oxygen species (ROS) generation [137,138]. During the pathogenesis of PD, ROS generation damages the substancia nigra through lipid peroxidation, protein oxidation and DNA oxidation [137,139]. According to the available research, this event seems to be induced mainly by changes in the brain iron content, mitochondrial dysfunction and monoamine oxidase activation, an enzyme responsible for dopamine metabolization [138,140,141]. There is also evidence that oxidative stress can induce α-synuclein conformational changes and increase its aggregation [142,143].

In 2008, Del Rio and Velez-Pardo demonstrated that CP-55,940, a non-selective cannabinoid receptor agonist, and JWH-015, a preferential CB2 receptor agonist, protect and rescue lymphocytes against paraquat exposition [28]. Paraquat is a Parkinson’s disease chemical inducer, and, in lymphocytes, induces mitochondrial damage and apoptosis through an oxidative stress mechanism involving ROS generation, namely superoxide anion radical (O_2_^−^), hydroxyl radical (OH) and hydrogen peroxide (H_2_O_2_) [144]. The authors showed that the abovementioned synthetic cannabinoids inhibit ROS formation, contributing to maintaining mitochondrial membrane potential and cell nucleus morphology [28]. According to this study, CP-55,940 and JWH-015 are protective due to potential anti-oxidative action. Subsequently, Velez-Pardo observed that CP-55,940 and JWH-015 attenuate paraquat-induced mitochondrial damage in the brain cortex of mice by scavenging O_2_^−^ and H_2_O_2_ and avoiding Ca^2+^-induced mitochondrial swelling, contributing to the maintenance of mitochondrial membrane potential [34]. The authors also revealed that JWH-015 has more effective and potent anti-oxidative effects when compared to CP-55,940. It is relevant to mention that CP-55,940 and JWH-015 exert an inhibiting effect against O_2_^−^ and do not cause any changes in mitochondrial membrane potential in mitochondria not exposed to paraquat [34].

##### Alzheimer’s Disease

AD is a neurodegenerative disease characterized by the deposition of β-amyloid peptide (Aβ) plaques and neurofibrillary tangles (NFT) of the protein tau [145,146]. AD is the most common type of dementia, a clinical syndrome that involves a progressive decline in two or more cognitive domains, including memory, language, executive and visuospatial function, personality and behaviour [147]. This disease is more commonly associated with elders, and its initial and most prevalent presenting symptom is episodic short-term memory loss with relative sparing of long-term memory [145,148]. This event is followed by impairment in problem-solving, judgment, executive functioning, lack of motivation, and disorganization, culminating in multitasking and abstract thinking difficulties [149]. Genetic and lifestyle factors, such as smoking and a series of pathologies and conditions, such as cerebrovascular diseases, diabetes, hypercholesterolemia and depression, seem to be involved in AD aetiology [149,150,151,152].

As previously mentioned, the abnormal presence of extracellular plaques of insoluble Aβ and flame-shaped NFT of the microtubule-binding protein tau in neuronal cytoplasm, especially in brain regions involved in memory processes, are the two main mechanisms in AD [146]. According to amyloid cascade theory, the cerebral accumulation of Aβ, particularly Aβ42 form, is the main event causing AD [153,154,155]. Considering that Aβ42 is generated through cleavage of the amyloid precursor protein (APP), reports indicate APP metabolism dysfunction with a subsequent increase in Aβ levels as a possible mechanism that promotes AD [155,156,157]. Additionally, it was already observed that abnormal Aβ plaques induce the phosphorylation of tau protein, which spreads via microtubule transport to neighbouring neurons, contributing to their death [158,159]. The molecular mechanisms are not fully understood yet. Still, the studies suggest that Aβ induces a series of processes that lead to abnormalities in tau protein folding, phosphorylation, degradation and localization, leading to neuronal and synaptic atrophy and death resulting from excessive stimulation of neurotransmitter receptors in neuronal membranes, collapse in calcium homeostasis, inflammation and depletion of energy and neuronal factors [147,149,158].

In 2009, Tolón et al. showed that JWH-015 induced the removal of Aβ-amyloid peptide from human frozen tissue section belonging to Alzheimer’s disease patients through the human macrophage cell line THP-1 [33]. It is worth mentioning that, without exposure to JWH-015, THP-1 macrophages cannot remove pathological deposits of Aβ-amyloid peptide.

Tolón and collaborators also showed that the JWH-015 effect is mediated by the CB2 receptor since adding SR144528, a CB2 receptor antagonist, cancels its effects in situ [33]. However, in vitro, the reversal of JWH-015 effects was not observed with the CB2 antagonist, which indicates a possible environment-dependent macrophage response. Through its action on CB2 receptors, JWH-015 exerts a stimulatory effect on the phagocytosis of Aβ-amyloid peptide by THP-1 macrophages, promoting its removal [33].

Concluding, the activation of the CB2 receptor by JWH-015 triggers the in situ phagocytosis of β-amyloid peptide by THP-1 macrophages, inducing its removal [33].

##### Neurocognitive Disorders Associated with HIV-I

Human immunodeficiency virus (HIV) is the causing agent of acquired immunodeficiency syndrome (AIDS), a chronic, potentially life-threatening condition caused by the human immunodeficiency virus [160]. This virus targets the immune system by destroying and impairing the function of immune cells, weakening the immunologic defences against a series of infections and pathologies [161]. Nowadays, anti-retroviral therapy (ART) is the available therapeutic option for AIDS. This therapy uses a set of medicines that target the enzymes reverse transcriptase, protease and integrase, among other vulnerable points in the HIV replication cycle [162]. Despite the considerable success of ART, HIV-I associated neurocognitive disorders, such as asymptomatic neurocognitive impairment, mild neurocognitive disorder and dementia, are still prevalent conditions without therapeutic options [163,164,165].

In 2013, Hu et al. demonstrated that WIN-55,212 attenuates neuronal damage and apoptosis caused by the HIV-1 gp120 protein in a human mesencephalic neuronal and glial culture model [166]. This protein induces neuronal damage and apoptosis, specifically in dopaminergic neurons, by decreasing DA uptake through the reduction in DAT function, causing morphological changes, inducing oxidative stress and reducing its viability, judging by a decrease in the number of dopaminergic neurons and a loss of dendrites [166]. The nigrostriatal dopaminergic area is a critical brain region for the neuronal dysfunction observed in HIV-1-associated neurocognitive disorders [167]. Interestingly, it has been shown that WIN-55,212 inhibits gp120-induced O_2_^−^ production, reducing oxidative stress. This effect was observed in the human mesencephalic culture exposed to gp120 alone and with purified human microglial cells that potentiate the neurotoxic effects of gp120 [166]. Hu and collaborators evaluated the capacity of WIN-55,212 to inhibit the migration of highly purified human microglia towards the supernatants generated from gp120-exposed human mesencephalic cultures. The authors found that the synthetic cannabinoid mentioned inhibits the migration of microglial cells, inhibiting the release of chemokines CCL2, CX3CL1, CXCL10 and cytokine IL-1β [128,166]. Finally, the CB2 receptor is the main one responsible for the effects of WIN-55,212 since its neuroprotection decreases more significantly in the presence of the CB2 antagonist SR144528 compared to the CB1 antagonist SR141716A [166].

#### 4.2.3. Oncologic Pathologies

Since phytocannabinoids’ anti-tumour properties discovery, several synthetic cannabinoids have been synthesized and subjected to research and trials as potential anticancer agents [168,169,170,171]. By interacting with cannabinoid receptors, synthetic cannabinoids can modulate crucial cellular signalling mechanisms and pathways for tumour development, including cell proliferation and survival [172]. As such, the antitumorigenic capacity of these compounds relies on the inhibition of tumour cell migration and proliferation, induction of apoptotic processes, reduction of tumour cell viability and blocking of angiogenesis and tumour invasion/metastasis [173,174]. The activation of these antitumorigenic processes by synthetic cannabinoids has been observed in several oncological studies, including multiple myeloma, osteosarcoma, glioblastoma multiforme and triple negative breast cancer [27,30,175,176].

##### Multiple Myeloma

Multiple myeloma is a clonal plasma cell proliferative blood disorder in which monoclonal plasma cells proliferate in bone marrow, leading to an overabundance of monoclonal paraprotein, destruction of bone and displacement of other hematopoietic cell lines [177,178]. Some common signs and symptoms include anaemia, bone pain or lytic lesions on X-ray, kidney injury and hypercalcemia, among others [179,180]. Despite its aetiology not being fully defined, the research on this topic indicates that environmental, lifestyle factors and genetic abnormalities in oncogenes, such as CMYC, NRAS and KRAS, are potentially critical for plasma cell proliferation [181,182].

In 2017, Barbado et al. demonstrated that WIN-55,212 reduces cell viability and induces selective apoptosis in multiple myeloma cell lines and spinal cord primary plasma cells of multiple myeloma patients while sparing normal cells from healthy donors, particularly hematopoietic stem cells [27]. This synthetic cannabinoid also suppresses tumour growth in mice [27].

These authors have shown that WIN-55,212 effects are mediated by apoptotic mechanisms, primarily through the activation of the initiator caspase caspase-2. Reinforcing this observation, in the presence of pan-caspase inhibitor Z-VAD-FMK, WIN55,212 pro-apoptotic effects are partially inhibited, showing that these effects are, at least in part, caspase-dependent [27].

To understand the process responsible for apoptosis induction, the authors evaluated the involvement of de novo synthesis of ceramides in the WIN-55,212-induced apoptosis. It was already demonstrated that ceramide is a potent suppressor that potentiates and drives the process of apoptosis [183]. It was observed that WIN-55,212 promotes the synthesis of ceramide through the upregulation of SPT, an essential enzyme in regulating ceramide synthesis [27,183]. The authors also demonstrated that WIN-55,212 also excerpts its effects through CB2 receptor activation since the CB2 receptor antagonists PGN-8, PGN-37 and PGN-70 blocked its action [27].

##### Osteosarcoma

Osteosarcoma is a malignant bone tumour characterized by osteoid production by malignant mesenchymal cells [184]. Its high degree of malignancy, strong invasiveness, rapid disease progression and extremely high mortality rate are associated with this pathology [185]. Osteosarcoma is the third most common cancer in adolescents [186]. Patients typically complain about localised and persistent pain usually noticed after an injury. Despite the patients being heavily treated for pain, the pain felt is never fully resolved [185,187]. Physical examination also allows observing warmth, skin vascularity or pulsations over the lesioned area [187]. Such as in multiple myeloma, osteosarcoma aetiology remains a poorly understood issue. However, available research indicates an important interplay of genetic and environmental factors, such as the exposition to certain types of radiation and chemicals, in osteosarcoma aetiology [184,188].

In 2019, Notaro et al. demonstrated that WIN-55,212 prevents cell migration and reduces extracellular levels of matrix metalloproteinases (MMP) 2 and 9 and drastically decreases intracellular levels of MMP9 in human osteosarcoma cell line MG63 [175]. MMP regulates many physiological and pathological processes, including normal tissue remodelling, angiogenesis, DNA replication, neurodegeneration and cancer [189]. This synthetic cannabinoid also prevents the release of secreted protein acidic and rich in cysteine (SPARC), inhibiting its secretion into the extracellular medium and promoting the upregulation of miR-29b1, a microRNA that inhibits cell proliferation and migration when overexpressed [175]. SPARC is involved in the regulation of cell adhesion and migration processes and tissue remodelling [190]. Notaro and collaborators also showed that the WIN-55,212-2 effects on cell migration are SPARC-independent and miR-28b1-dependent since, when SPARC expression is silenced in MG63 cells, by RNA interference, WIN55,212 continues to reduce cell migration [175]. Conversely, a reduction in cell migration is observed in cells transfected with miR-29b1 and treated with WIN-55,212 but not in cells not transfected with this microRNA. These results demonstrate the importance of WIN-55,212-induced miR-29b1 upregulation to its anti-migratory effects [175].

##### Glioblastoma Multiforme

Glioblastoma multiforme is a primary malignant CNS tumour that arises from astrocytes [191,192]. Astrocytes are a sub-type of glial cells located in the CNS that provide physical and metabolic support to neuronal cells, including neuronal communication, nutrient supply and waste removal [193]. This oncologic pathology is the most prevalent, aggressive and invasive CNS tumour in adults [192,194]. Patients with glioblastoma multiforme can exhibit several symptoms, such as headaches, seizures, memory loss and functional impairment [195]. The aetiology of glioblastoma multiforme is poorly understood, the exposure to high dose ionizing radiation being the only aetiologic possibility confirmed [196]. Nevertheless, genetic and environmental factors in glioblastoma multiforme aetiology are an object of research [191].

In 2012, Gurley et al. found that KM-233, a non-selective CB1 and CB2 receptor agonist, causes a time-dependent change in the phosphorylation profiles of MEK, ERK1/2, Akt, BAD, STAT3 and p70S6K in the glioblastoma multiforme human cell line u87MG [30]. This synthetic cannabinoid also promotes a redistribution of the golgi–endoplasmic reticulum structures and an almost complete mitochondrial depolarization by a rapid increase in cleaved caspase 3 levels and significant cytoskeletal contractions, which are indicators of apoptosis [30]. The alterations in mitochondrial membrane polarization lead to mitochondrial integrity loss and the formation of autophagic compartments and vacuoles. The formation of these structures is an indicator of autophagy, evidencing the initiation of a mitochondrial-mediated autophagy process [30]. Additionally, KM-233 promotes an 80% reduction in tumour size by decreasing its growth rate without showing acute toxicity in the mice organs [30]. KM-233 effects are inhibited in the presence of SR141716A, a CB1 receptor antagonist, suggesting CB1 receptor involvement in the process. Concluding, KM-233 may be a potential treatment for glioblastoma multiforme cases through its pro-apoptotic and anti-proliferative properties [30].

##### Triple Negative Breast Cancer

Triple-negative breast cancer is a sub-type of breast cancer defined by the lack of expression of the three principal biomarkers associated with breast cancer: oestrogen receptor-α, progesterone receptor and human epidermal growth factor receptor 2 (HER2) [197]. These characteristics lead to a poorer prognosis and a reduced response to therapeutics, making this sub-type of breast cancer more aggressive and fatal [198]. Breast cancers represent the most common cancers diagnosed in women [199]. From this, about 10 to 15% correspond to triple-negative breast cancer [200]. The typical symptoms are similar to those observed in other forms of breast cancer, including partial or complete breast swelling, breast or nipple pain, nipple retraction and swollen lymph nodes [201]. Genetic factors, such as mutations in BRCA1 and BRCA2 genes, are key for triple-negative breast cancer aetiology [202,203]. These gene products act as cell growth suppressors, playing a protective role against tumorigenesis [204]. The evidence shows that mutations in these genes promote an increased risk of developing breast cancer [205].

In 2018, Greish et al. demonstrated that SMA-WIN, a nanomicellar formulation of WIN55,212, reduces tumour growth by promoting necrotic areas in the tumours in mice with triple-negative breast cancer 4T1 [176]. In addition, this formulation reduces the psychoactive effects of WIN-55,212 free form. The lower psychoactive effect is due to a significantly lower concentration of micelles in the brain when compared to the WIN-55,212 free form concentration, suggesting that blood–brain barrier permeability to the nanomicellar structure is reduced [176]. The studies indicate that the formulation has reduced blood–brain permeability and a propensity to accumulate in the tumour site [176].

#### 4.2.4. Other Pathologies and Conditions

Acute and chronic inflammation underly so many diseases that it is hard to pinpoint all the pathologies involving inflammatory processes. According to Philip Hunter, it would make sense to recognize inflammation as a condition that should be treated in its own right, “to make possible the development of a new generation of drugs to treat conditions including cancers, autoimmune disorders and infectious diseases” [206]. Thus, cannabinoids and synthetic cannabinoids research may make possible the development of a new generation of drugs to treat conditions, including those already mentioned and others that do not fit in the previous categories.

##### Trigeminal Neuralgia

In 2007, Liang et al. found that WIN-55,212 increases the mechanical response threshold and the latency response to heat stimulation in mice with trigeminal neuropathic pain caused by a chronic constriction injury in the infraorbital branch of the trigeminal nerve [207]. However, a lower dose of WIN-55,212 increased the mechanical response threshold without modifying the thermal response threshold, revealing that this synthetic cannabinoid is more potent against mechanical allodynia than thermal hyperalgesia [207]. It is important to note that, excepting the highest concentration tested (5 mg/kg), WIN-55,212 promoted the above-mentioned effects without causing motor deficits or body temperature changes in the mice.

Using CB1 and CB2 receptor antagonists AM-251 and AM-630, respectively, it was demonstrated that WIN-55,212 effects are mediated through its action on the CB1 receptor since AM-251, but not AM-630, completely reversed the antiallodynic and antihyperalgesic WIN-55,212 effects [207].

##### Cognitive Dysfunctions—Recognition Memory

In 2011, Bialuk and Winnicka demonstrated that AM-251, a preferential CB1 receptor antagonist/reverse agonist, improves recognition memory by improving mice’s ability to acquire and consolidate information without provoking anxious behaviour and changes in psychomotor capacities [208]. It is relevant to mention that the desirable effects were seen with the lowest concentration tested (1.0 mg/kg), while the higher concentrations (2.5 mg/kg and 5.0 mg/kg) did not influence recognition memory.

To understand how AM-251 exerts its pro-cognitive effects, Bialuk and Winnicka put forward three possible mechanisms of action: (i) CB1 receptor-mediated endocannabinoids inhibition of the release of acetylcholine in the neocortex leading to a decrease in cholinergic transmission promoting memory deficits; (ii) activation of GPR55 receptor followed by cellular calcium mobilization by AM251 due to GPR55 expression in the ventral hippocampus, critical for cognition and recognition memory; (iii) AM-251 inverse agonistic/antagonistic properties on CB1 receptor [208,209,210,211].

##### Accelerated Gastrointestinal Motility

In 2015, Keenan et al. found that AM-841 reduces intestinal and colonic motility in mice under physiological and acute stress conditions [212]. However, AM-841 is more potent in acutely stressed mice than normal stress-accelerated gastrointestinal motility. According to this study, the AM-841 effects are present in mice with CB1 and CB2 receptors and in mice knocked out for CB2 receptors [212]. However, the AM-841 effects are abolished in mice knock out for CB1 receptor, suggesting the involvement of the CB1 receptors present in the small and large intestines. According to this study, AM-841 reduces gastrointestinal motility in mice through CB1 receptors in the intestinal level in acutely stressed mice [212].

##### Peripheral Inflammation and Tissue Repair

In 2016, Bort et al. found that JWH-015, a preferential CB2 receptor agonist, reduces the concentration of pro-inflammatory factors, such as interleukin-6 (IL-6) and monocyte chemoattractant protein-1 (MCP-1), and increases the concentration of anti-inflammatory factors, namely transforming growth factor β (TGF-β), in keratinocytes and fibroblasts treated with lipopolysaccharide, an inflammatory stimulus [213]. Keratinocytes and fibroblasts are two of the main cell types that, through activation mediated by inflammatory signals such as pro-inflammatory factors, respond to the inflammatory phase in skin repair, essential for wound healing [214,215]. The observed effects were promoted by CB1 and CB2 receptors, except the decrease in MCP-1 concentration in the keratinocytes. This reduction was promoted by the action of JWH-015 on the CB2 receptor since the CB1 receptor antagonist AM-281 did not decrease or nullify the effect observed [213].

Additionally, Bort and collaborators evaluated the rate of permeation of JWH-015 using swine skin. The authors showed that JWH-015 is mostly retained in the skin and displays a sustained and low-level transdermal permeation, revealing that this synthetic cannabinoid can reach the pretended target [213].

##### Pulmonary Fibrosis

In 2016, Lucattelli et al. demonstrated that ajulemic acid, a preferential CB2 receptor agonist, significantly reduces the fibrotic response in the inflammatory and early fibrogenic phase in mice with bleomycin-induced pulmonary disease fibrosis [216]. The authors observed a reduction in the number of inflammatory cells, an attenuation of collagen deposition destruction in lung architecture and a decrease in the fibrotic areas [216]. Additionally, this synthetic cannabinoid induces changes in the expression pattern of products involved in fibrogenesis, namely TGF-β1, pSMAD2/3, CTGF and α-SMA. Ajulemic acid reduces the expression, tissue levels and retention of these indicators of myofibroblastic differentiation, leading to a decrease in the number of active fibrogenic cells [21].

Lucattelli and collaborators also found that ajulemic acid increases the number of PPAR-γ positive cells and its prevalence at the nuclear compartment, even with a low grade of fibrosis [216]. PPAR-γ (proliferator-activated receptor γ) is a ligand-inducible transcription factor that belongs to the nuclear hormone receptor family [217]. This transcription factor is involved in a series of biological processes, including modulation of inflammatory and immune response, cell differentiation and wound healing, these last two events being related to fibrogenesis [217,218]. It is also important to mention that it was already demonstrated that ajulemic acid binds specifically to PPAR-γ, promoting its transcriptional activity [219].

According to this study, ajulemic acid limits the progression of bleomycin-induced pulmonary fibrogenesis through its anti-inflammatory and anti-fibrotic properties. The authors suggest that the anti-fibrotic ajulemic acid effects are mediated through PPAR-γ action [216].

##### Seizures and Epilepsy

In 2017, Huizenga et al. demonstrated that WIN-55,212 and ACEA, a preferential CB1 receptor agonist, exhibit anticonvulsant effects against clonic seizures, tonic–clonic seizures and both chemoconvulsant-induced and acute hypoxia-induced seizures in mice [220]. Additionally, these synthetic cannabinoids increase the time interval between clonic seizure stimulation and its emergence (latency to clonic seizures) and protect mice against tonic extension in tonic–clonic seizures [220]. However, it is important to note that, at the concentrations required to observe the anticonvulsant effects, WIN-55,212 provokes significant sedative effects. The CB2 and GPR55 receptors agonists and the CB2 receptor antagonists HU-308, O-1602 and AM-630, respectively, showed no effect [220].

Interestingly, the anticonvulsant effects previously mentioned were only observed in P10 mice. P10 corresponds to an age stage equivalent to newborns in humans [220].

Huizenga and collaborators showed that the WIN-55,212- and ACEA-induced decrease in seizures severity is mediated by their action on the CB1 receptor since the CB1 receptor antagonist AM-251 reverses this effect [220]. However, AM-251 exhibits no effect on clonic or tonic–clonic seizures latency.

Considering these results, the authors suggest that the anticonvulsant action of the cannabinoid receptor is due to CB1 receptor activation during early development. Moreover, they emphasise the therapeutical potential of WIN55,212 and ACEA as antiepileptic drugs for epilepsy in infants and children [220].

In 2020, Griffin et al. demonstrated that JWH-018 N-(5-chloropentyl) analogue, JWH-018 N(2-methylbutyl) isomer, 5-fluoro PB-22 5-hydroxyxisoquinoline isomer, 5-fluoro ADBICA and AB-FUBINACA 3-fluoronezil isomer exhibit anticonvulsant activity by decreasing seizure-like swim behaviour and electrographic seizures frequency in a zebrafish model with Dravet syndrome [221]. Electrographic seizures correspond to seizures that are evident in electroencephalographic monitoring and are common in children and newborns with encephalopathy in an advanced stage [221].

##### Intestinal Ischemia and Reperfusion

In 2019, Bayram et al. demonstrated that AM-1241, a preferential CB2 receptor agonist, allows the recovery of smooth ileum muscle functional contractility in mice with intestinal ischemia and reperfusion [222]. This improvement was accompanied by a decrease in malondialdehyde (MDA) levels, a reverse in the depletion of glutathione (GSH) levels observed in mice with intestinal ischemia and reperfusion, inhibition of pro-inflammatory factors expressions, such as TNF-α and IL-1β, and a decrease in myeloperoxidase (MPO) activity in the intestinal tissue [222]. MPO is a heme-containing peroxidase expressed in immunocytes that, in the presence of hydrogen peroxide and halides, catalyses the generation of reactive oxygen intermediates. Its activity is related to cell migration and is increased in intestinal ischemia and reperfusion, contributing to oxidative stress [223]. Moreover, MDA is one of the final products of lipid peroxidation, for which an increase in ROS stimulates overproduction. As such, MDA is an indicator of oxidative stress [224].

Bayram and collaborators also showed that the AM-1241 anti-inflammatory and oxidative stress preventing effects are mediated through its action on the CB2 receptor since its antagonist, JTE-907, reverses the effects previously mentioned [222].

##### Traumatic Brain Injury

Relative to HU-910, it was observed that this synthetic cannabinoid increases synaptogenesis by promoting the increase in the synaptophysin protein in the brain cortical cytosolic fraction, which indicates a higher synaptic density [225]. Synaptogenesis is a process characterized by synapses/connections forming between the neurons [226]. Regarding HU-914, this compound promotes neurological recovery, as seen through a decrease in the injured brain area and an increase in the number of axons in the corticospinal tract, and inhibits the release of pro-inflammatory TNF-α cytokine in the ipsilateral hippocampus [225].

Additionally, Magid and collaborators have shown that the HU-910 and HU-914 effects are mediated by the CB2 receptor, judged both by the inhibition of its effects in the presence of CB2 receptor antagonists SR144538 and AM630, HU-910 and in CB2 knocked out mice [225].

##### Brain Injury

In 2020, Al-Eitan et al. demonstrated that XLR-11, a non-selective cannabinoid receptors agonist, enhances the viability of human brain microvascular endothelial cells (HBMECs) by increasing the number of viable cells [227]. Additionally, this synthetic cannabinoid increases the migration rate of brain endothelial cells, the first stage in the angiogenesis process, and promotes their angiogenic capacity. [227] Angiogenesis is a biological process characterized by the formation of new blood vessels through pre-existent blood vessels. This process plays a pivotal role in a series of events, such as tissue growth and wound healing [228]. This was observed through an in vitro tube formation assay that revealed an increase in the number of formed tubular structures, in their total length, in the number of branching points and the number of loops [227].

In conclusion, XLR-11 promotes cell viability in HBMECs and enhances angiogenesis in the brain in vitro. Considering these results, Al-Eitan and collaborators suggest that this synthetic cannabinoid has the potential to be used as a therapeutic angiogenic drug in cases of human brain injury [227].

##### Type 1 Diabetes

In 2021, Hove et al. found that JWH-133, a preferential CB2 receptor agonist, promotes endothelial (eNos)- and neuronal (nNOS)-dependent dilation of cerebral arterioles in mice with type 1 diabetes [229]. This synthetic cannabinoid also improves brain arterioles’ response to ADP, an eNOS-dependent agonist, and NMDA, an nNOS-dependent agonist, contributing to increased cerebral arterioles dilation. The authors have shown that JWH-133 effects are mediated by the CB2 receptor since its antagonist AM-630 inhibits the effects mentioned above [229].

Overall, the study suggests that JWH-133 can act as a potential therapeutic agent for treating cerebral vascular diseases via a mechanism that promotes the increase of cerebral blood flow [229]. Concerning the mechanism through which this synthetic cannabinoid improved cerebral arterioles dilation in T1D mice, based on the available literature, Hove and collaborators hypothesised that JWH-133 may influence oxidative stress, stimulate the PPARs pathway in brain arterioles or inhibit the synthesis and release of inflammatory mediators [229,230,231].

## 5. Toxicity of Synthetic Cathinones and Synthetic Cannabinoids

### 5.1. Synthetic Cathinones

Synthetic cathinones were primarily synthesized for therapeutic purposes. However, all synthetic cathinones, excepting bupropion, branded as Wellbutrin [41,81], and amphepramone, branded as Tenuate and Hipofagin, were withdrawn from the market due to vast cases of dependency and associated toxicity [69].

Synthetic cathinones produce distinct pharmacological effects, with psycho-stimulation and hallucinations. Taking the effects of mephedrone, for example, they are simultaneously psychostimulant (i.e., like amphetamine) and hallucinogenic (like MDMA). Therefore, synthetic cathinones and amphetamines share pharmacological properties, and, based on the extensive similarities in the effects of these drug classes, it might be predicted that these cathinones would cause neurotoxicity to DA and 5-HT nerve endings identical to methamphetamine METH and MDMA [68,232]. Moreover, overdose can occur after continuous daily consumption and can lead to psychological illness with paranoid or delusional mania symptoms. Finally, withdrawal syndrome was reported after suspension and was characterized by insomnia, lack of concentration, craving, nightmares and slight trembling [233]. In addition to the neuropsychiatric symptoms, consumers often present with sympathomimetic toxicity. Consistently, several cathinones have been associated with the development of serotonin syndrome, eventually leading to fatalities [234]. Therefore, some studies have been developed to elucidate the mechanisms underlying the synthetic cathinones toxicity [232,235]. The toxicities of the synthetic cathinones with medical potential are described in this section.

#### 5.1.1. Molecular and Cellular Studies

The neurotoxic profiles of methcathinone, metamphepramone and amphepramone were accessed in a human neuronal cell line (differentiated neuroblastomas SH-SY5Y) [236]. Firstly, the potential toxicity of thirteen synthetic cathinones was studied, and metamphepramone and amphepramone were the least toxic [236]. Furthermore, these substances promoted apoptotic death and intracellular vacuolization, and methcathinone triggered autophagy activation.

Pyrovalerone is used as a medicine. However, there were no studies found relating to toxicity results for pyrovalerone. Nevertheless, α-PVP is a synthetic cathinone used as a recreational drug that shares chemical structure similarity with pyrovalerone. In the study of Soares et al., this compound showed to be one of the most toxic synthetic cathinones addressed [236]. Moreover, α-PVP was also shown to be myotoxic by impairing the cell membrane integrity, depleting ATP levels and increasing mitochondrial superoxide concentrations in a myoblasts cell line (C2C12) [237]. On the other hand, MDPV showed similar effects to α-PVP, also being myotoxic [237]. Moreover, this synthetic cathinone was also shown to be hepatotoxic [238].

#### 5.1.2. Animal Studies

Most cathinone derivatives increase locomotor activity in rodent models at a similar dose range. Methcathinone and bupropion displayed reduced immobility time, suggesting antidepressant potential [239]. In rats, it has also been verified that cathinone and its synthetic forms led to behavioural sensitization [233]. Repeated administration of methcathinone was neurotoxic to the central dopaminergic system by reducing the number of DA transporter sites, central 5-HT and DA content and the activity of tryptophan hydroxylase (TPH) and tyrosine hydroxylase (TH), both responsible for the synthesis of 5-HT and catecholamines, respectively. On the other hand, the MDPV consumption did not decrease the levels of TH, DA or DAT [232,240]. However, long-term use of MDPV showed to increase the risk of neurocognitive dysfunction and neurodegeneration in the prefrontal cortex or hippocampus [241].

#### 5.1.3. Case Reports from Emergency Rooms

According to the US National Association of Pharmacy Boards, synthetic cathinones were linked to an estimated 22,904 visits to hospital emergency rooms in 2011 [242]. This tendency seems to have risen worldwide judging by a recent analysis of the number of case reports of patients taking a series of new psychoactive substances [243]. The symptoms described by clinicians include hyperthermia, agitated delirium, tachypnoea, coagulopathy, rhabdomyolysis and cardiac and other organs arrest or failure. In several cases, other psychoactive drugs have been detected in deceased patients [244]. Fatal cases related to the consumption of synthetic cathinones have been reported. However, many of these cases are associated with exposition to synthetic cathinones concomitantly with other drugs of abuse [45,73,244]. In this section, reported fatal toxicity cases after exposition to synthetic cathinones, therapeutically used, are addressed.

The recommended amphepramone intake is 75 mg/day [245], and the adverse effects reported are urticaria, rash, ecchymosis and erythema [246]. A 52-year-old woman went to the hospital with a rash suggestive of cutaneous vasculitis. She reported the ingestion of an ‘herbal medicine’ for weight reduction. Samples of the supplement were analysed, and it was determined that each tablet contained 69 mg of amphepramone. After four weeks of taking this supplement twice daily, the patient suffered from flu-like symptoms of varying intensity for one week, and, one month later, the itching started. Dark-red, painful, palpable skin lacerations and central necrosis appeared symmetrically on both legs.

Methcathinone hydrochloride, after being intravenously injected, promoted a new form of alleged manganese poisoning [247]. A case report on manganese abuse revealed eight patients abusing methcathinone intravenously, all of them for at least five months. The reported clinical symptoms were balance difficulties, dysarthria, apraxia as well as extrapyramidal symptoms (bradykinesia, hypomimia, facial dystopia, micrography). Laboratory analyses showed elevated serum Mn levels exceeding 2.0 μg/L (normal range 0.3–0.9 μg/L) in all the patients [248].

No reported cases were found for pyrovalerone. However, there are fatal cases reported from two pyrovalerone derivatives, α-pyrrolidinohexiophenone (α-PHP) and α-PVP [249].

The first reported fatal overdose with MDPV, resulting from a cardiac arrhythmia, occurred in 2012 [250,251]. This synthetic cathinone can cause multi-organ collapse. Patients with MDPV intoxication and concomitant renal injury seem to profit from haemodialysis [252]. Repeat intoxication events may produce acute renal injury full of metabolic derangements, including metabolic acidosis, hyperuricemia and rhabdomyolysis [93,253].

Bupropion is also reported in the literature. There are reported cases of serotonin toxicity involving recreational use of bupropion [254], either on its own [255,256] or in combination with other serotonergic medications. These cases led to bupropion notification as an NPS in 2014 [257]. In a report regarding cases of serotonin toxicity, from information collected in an international database between 2010 and 2016, antidepressants were the most common cause of serotonin syndrome, with bupropion being the most frequent overall [258].

Metamphepramone consumption is not associated with hospital emergency cases.

There are many other case reports in synthetic cathinones consumption for which therapeutical studies were not found [239,244,259,260,261,262,263,264,265].

### 5.2. Synthetic Cannabinoids

Despite their potential therapeutic use in a wide variety of pathologies and conditions, synthetic cannabinoids are associated with toxicity and several adverse effects [266,267,268,269,270,271,272,273,274,275]. Focusing on the synthetic cannabinoids that demonstrated medical potential, namely those belonging to the JWH, HU and AM series, the present section describes the toxicological effects observed.

#### 5.2.1. Molecular and Cellular Studies

Some studies explore the toxic effects at the molecular and cellular levels in the literature. These studies are usually performed in several models, namely carcinogen and non-carcinogen cells, and it becomes challenging to compare between them. Therefore, the studies were divided between cell lines.

##### Neuronal Human Cells

In 2016, Wojcieszak et al. demonstrated that synthetic cannabinoid JWH-133 significantly decreases cell viability and proliferation, depending on the concentration, in SH-SY5Y human neuroblastoma cells [266]. Moreover, JWH-018 and JWH-122 also promote a decrease in cell viability by lysing cell membranes [266]. This lower viability was also seen in other cell models, such as choriocarcinoma cells (BeWo) [267], epithelium cells (TR146), breast derived cells (MCF-7) and TK6 lymphoblastoid cells [268]. Moreover, the cellular mechanism underlying this effect was explored. Sezer and collaborators observed an increment in oxidative stress by increasing MDA levels, decreasing the activity of glutathione reductase (GR) and catalase, responsible for the detoxification of peroxide hydrogen (H_2_O_2_), reducing glutathione levels (GSH), a known endogenous antioxidant that protect cells from oxidative reactions [269]. Based on these results and considering that studies indicate that JWH-133 has therapeutic potential, the authors call for caution in its possible use as this substance may accumulate in the CNS and lead to neuronal damage [266]. Contrastingly, in the same cellular model, Couceiro et al. and Sezer et al. found that synthetic cannabinoid JWH-018 does not significantly modify the cell viability of SH-SY5Y cells [269,270]. Later, using yeast as a model, it was observed that this synthetic cannabinoid increases the rate of cell proliferation significantly through an increase in glycolytic flow to the detriment of the pentose phosphate pathway, which suffers a decrease [271]. More studies have demonstrated that JWH-018 is not toxic for cells, neither by genotoxicity nor promoting apoptotis/necrotis [269]. Furthermore, these results were corroborated in other cell lines, such as in the epithelium (TR146)- and breast (MCF-7)-derived cell lines [272]. However, there are also contradictory results in these cell lines since Koller et al. observed genotoxicity in the epithelium (TR146)- and breast (MCF-7)-derived cell lines after exposure to JWH-073, JWH-122 and JWH-210 [275].

However, synthetic cannabinoids metabolization originates metabolites that can be more toxic than parent compounds. Couceiro and collaborators determined that, contrastingly to JWH-018, its metabolite N-(3-hydroxypentyl) promotes a decrease in cell viability, lysis of the cell membranes and consequent death via necrotic processes. It is essential to mention that all the observed results in SH-SY5Y cells also occurred in kidney human embryonic cells HEK293T [270]. According to these results, the authors showed that phase I metabolites of JWH series synthetic cannabinoids may have biological activity, highlighting the importance of their toxicological profile to understand the long-term adverse effects of these substances [270].

##### Mice Neural Cells

In 2011, Tomiyama and Funada showed that synthetic cannabinoids CP-55,940, CP-47,497 and CP-47,497-C8 promote apoptosis in mice neuroblastoma and glial cells (NG 108-15) by inducing the activation of caspase 3 [273]. Morphological changes related to cytotoxicity were observed, namely nuclear fragmentation and condensation, which lead to neuronal damage. Moreover, the CB1 receptor antagonist AM-251 supressed the observed cytotoxicity, showing that the CB1 receptor has a pivotal role in synthetic cannabinoid-induced toxicity [273]. The referred observations were corroborated in mice forebrain cells [274]. They also observed that CP-55,940, CP-47,497 and CP-47,497-C8 reduce cell viability. Moreover, other synthetic cannabinoids were tested, namely JWH-018, JWH-210, HU-210 and AM-2210, which showed similar effects [274]. Cha et al. also used the mice forebrain cells model to study JWH-081 and JWH-210. Its exposition decreased the number of neuronal cells and distortion of the nuclei and respective nuclear membranes throughout the entire length of the central zone of nucleus accumbens, corroborating its neurotoxic properties [275].

Considering the presented studies, it is quite challenging to gather strong evidence since different synthetic cannabinoids and cellular models are used. Various parameters are evaluated, and, even with the same substance and model, sometimes, contradictory results are obtained. Nevertheless, it is possible to observe a prevalence in reduced cell viability and apoptosis induction, even in non-carcinogenic cells.

#### 5.2.2. Case Reports from Emergency Rooms

The adverse effects promoted by synthetic cannabinoids are observed mostly at the neurological and psychiatric levels and in the cardiovascular and gastrointestinal systems [276,277]. In acute intoxication, the adverse effects include tachycardia, agitation, drowsiness, confusion, nausea and vomiting and hallucinations [277,278]. In most of the clinical cases recorded, the listed effects are not life-threatening and cease six to eight hours after consumption [279]. Before severe intoxication with these substances, the main manifestations identified are chest pain, myocardial infarction, acute kidney damage, seizures, acute psychosis, panic, hallucinations and paranoia, which may culminate in death attributed directly and indirectly to this set of synthetic cannabinoids [51,55,276,280]. The cases of death attributed directly to these compounds result from dysrhythmia, seizures and multiple organ failure, while deaths attributed indirectly to these cannabinoid receptor agonists come from hypothermia, development of trauma and self-mutilation [277,280,281]. Compared to ∆^9^-THC, exposure to synthetic cannabinoids increases the severity and intensity of adverse effects [56]. In this context, it was concluded that the risks of agitation and cardiotoxicity, promoted by these synthetic cannabinoids, are 3.8 and 9.2 times higher than after traditional cannabis use, respectively, and that the likelihood of the consumer to resort to emergencies due to the adverse effects suffered is 30 times higher [282,283].

In addition to the systems previously mentioned, the clinical manifestations triggered by synthetic cannabinoids are also identified at the neuromuscular, neurobiological and metabolic levels, respiratory and renal systems, eyes and mouth [276]. At the neuromuscular level, myoclonus may occur, a manifestation characterized by involuntary, brief and sudden muscle contractions and increased creatine kinase (CPK) enzyme levels, resulting from the destruction of muscle tissue [24,276]. At the metabolic level, it may verify hyperglycaemia) and hyponatremia [280]. Dyspnea and tachypnea can be identified in the respiratory system, and, in the renal system, urine production-related and renal failure-related dysfunctions may occur [282,283]. In the ocular system, mydriasis and weak reaction of the pupils to light exposure may occur, and the consumer may also experience a sense of dry mouth (xerostomia) [24,55]. In addition to the manifestations associated with the nervous system, after consuming synthetic cannabinoids, the consumer may exhibit headaches, irritability, sensitivity to light, cognitive difficulties and anxiety and, at the cardiovascular level, hypertension, palpitations, dysrhythmia and chest pain [38,55,279,280].

The use of these substances, through one or repeated exposures, may trigger an acute psychotic reaction in healthy subjects and lead to effects that mimic symptoms characteristic of a clinical diagnosis of schizophrenia, such as changes in perception, depersonalization, development of a dissociative state, auditory and visual hallucinations, disorganized behaviour and discourse and suicidal ideation [52,54]. These symptoms may manifest more intensely in individuals with an established diagnosis of psychotic pathology [52]. The effects previously mentioned and the synthetic cannabinoids associated can be seen in Table 1.

## 6. Synthesis of the Available Data

The present work addresses the current knowledge of the therapeutic potential and toxicity of NPS, namely synthetic cathinones and synthetic cannabinoids, the most used according to the EMCCDA. 

### 6.1. Synthetic Cathinone

As stimulants, synthetic cathinones, particularly methcathinone, were medically used to treat depression during the 1930s and 1940s. However, methcathinone was removed as a therapeutic option due to its side effects. Therefore, pharmacological research allowed the medical approval of bupropion for depression and smoking cessation and pyrovalerone for chronic fatigue. Other synthetic cathinones were developed but were removed from the market or did not pass in a clinical trial, such as methcathinone for depression, and amphepramone and metamphepramone for obesity. MDPV was reported as being used illegally by ADHD adolescents. The misuse of these stimulants draws a thin line between prescribed and non-prescribed exposition to the substances. It was a small leap from this to the introduction of synthetic cathinones as recreational substances on the market. This was, probably, the first contemporary step between the therapeutical use and recreational use of psychoactive substances. 

### 6.2. Synthetic Cannabinoids

Synthetic THC medicine (dronabinol) and a synthetic THC analogue (Nabilone) are approved as antiemetic and analgesic medication in the US and Canada. Furthermore, several synthetic cannabinoids are being studied mainly for inflammatory, neurodegenerative and oncological diseases. CP-55,940, HU-308 and AM-841 are under research investigation for inflammatory conditions. Both inflammation reduction and immune system modulation were observed, which are promising results. The NPS research on neurodegenerative diseases is mainly focused on synthetic cannabinoids. Moreover, CP-55,940, JWH-015 and WIN-55,212 have also been studied. Furthermore, JWH-015 showed therapeutical potential by triggering phagocytosis of the β-amyloid peptide by THP-1 macrophages. WIN-55,212 and KM-233 have been extensively studied in cell models of oncological diseases. WIN55,212 showed CB2 receptor-mediated selective anti-myeloma and pro-apoptotic properties, and KM-233 showed therapeutical potential due to its pro-apoptotic and anti-proliferative properties. Interestingly, a nanomicellar formulation of WIN55,212 presented the same anti-myeloma and pro-apoptotic properties as the free form without the secondary psychoactive effects. Fourteen other synthetic cannabinoids are being studied for several other conditions. Although targeting the cannabinoid system seems to be a promising therapeutic approach, cannabis-based drugs interact with receptors other than CB receptors, having unexpected outcomes in clinical studies compared to preclinical trials. Considering the presented studies, it is difficult to make sense of the overall data since different cellular models are used, various parameters are evaluated and, sometimes, contradictory results are obtained even with the same substance and model. Nevertheless, a prevalence in reduced cell viability and apoptosis induction is observed, even in non-carcinogenic cells. Actually, the poor understanding of the endocannabinoid system, namely the mechanism of action of cannabinoids on other receptors besides CB1 and CB2, is hindering this field’s development. Further studies on the less known receptors of the endocannabinoid system are paramount for the therapeutic use of synthetic cannabinoids. 

### 6.3. Final Remarks 

Another issue in cannabinoids and cathinones research is their psychoactive nature. This property makes their recreational use desirable, posing a societal challenge to the frame used to control, or not, such substances. Since the 1961 and 1971 UN Conventions, the repressive control imposed by countries seems outdated presently. During the last 50 years, the scientific achievements may allow us to draw another control frame. Accordingly, the control due to health hazards seems to be the most promising framework. The relationship between the costs and benefits posed to pharmacological drugs should be applied to NPS. Simultaneously, education of individuals on the effects of NPS or other chemicals should be included in the educational programs of teenagers and young adults. This new paradigm may change chemical therapeutic research and the NPS trend balance from a razor’s edge to a wider road.

## Figures and Tables

**Table 1:** Examples of clinical case, registered in Europe and USA, associated with the consumption of synthetic cannabinoids of the JWH series and respective symptomatology, in various systems. (Adapted from Tournebize et al., 2016).

	Affected System					
Drug of Abuse	Nervous	Cardiovascular	Digestive	Respiratory	Ocular	Country
JWH-018 JWH-122	Headaches, confusion, visual and auditory hallucinations, irritability	Tachycardia	Vomiting	-	Mydriasis and slow reaction to light	Italy
JWH-018 JWH-073	Seizures, anxiety, paranoia	Tachycardia and hypertension	Abdominal pain, nausea, vomiting	Tachypnea	Mydriasis	USA
JWH-018 JWH-081 JWH-250	Seizures and confusion	Hypertension	Vomiting	-	-	USA
JWH-018	Cognitive impairment, insomnia, depression, twitching, dependency	Tachycardia, hypertension, palpitations	Nausea, diarrhea	-	-	Germany
JWH-210	Disturbance, anxiety, panic attacks and sedation	Tachycardia, hypertension	Vomiting, diarrhea, intense thirst	Dyspnea	Mydriasis	Germany

## References

[1] Specka M., Kuhlmann T., Sawazki J., Bonnet U., Steinert R., Cybulska-Rycicki M., Eich H., Zeiske B., Niedersteberg A., Schaaf L. (2020). Prevalence of Novel Psychoactive Substance (NPS) Use in Patients Admitted to Drug Detoxification Treatment. Front. Psychiatry.

[2] United Nations Office on Drugs and Crime. https://www.unodc.org/documents/scientific/NPS_leaflet_2018_EN.PDF.

[3] King L.A., Kicman A.T. (2011). A brief history of “new psychoactive substances”. Drug Test. Anal..

[4] United Nations Office on Drugs and Crime. https://www.unodc.org/LSS/Page/NPS.

[5] European Monitoring Centre for Drugs and Drug Addiction. https://op.europa.eu/en/publication-detail/-/publication/7bd409b6-44cd-11eb-b59f-01aa75ed71a1/language-en.

[6] Hosztafi S. (2001). The history of heroin. Acta Pharm. Hung..

[7] International Narcotic Control Board. https://www.incb.org/documents/Narcotic-Drugs/1961-Convention/convention_1961_en.pdf.

[8] Edeleano L. (1887). Ueber einige Derivate der Phenylmethacrylsäure und der Phenylisobuttersäure. Berichte Dtsch. Chem. Ges..

[9] Anderson E.W. (1938). Further observations on benzedrine. Br. Med. J..

[10] Rasmussen N. (2008). America’s first amphetamine epidemic 1929–1971: A quantitative and qualitative retrospective with implications for the present. Am. J. Public Health.

[11] Harris S.C., Ivy A.C., Searle L.M. (1947). The mechanism of amphetamine-induced loss of weight. J. Am. Med. Assoc..

[12] International Narcotic Control Board. https://www.incb.org/documents/Psychotropics/conventions/convention_1971_fr.pdf.

[13] Passie T. (2018). The early use of MDMA (‘Ecstasy’) in psychotherapy (1977–1985). Drug Sci. Policy Law.

[14] Grinspoon L., Bakalar J.B. (1986). Can Drugs Be Used to Enhance the Psychotherapeutic Process?. Am. J. Psychother..

[15] Mithoefer M.C., Grob C.S., Brewerton T.D. (2016). Novel psychopharmacological therapies for psychiatric disorders: Psilocybin and MDMA. Lancet Psychiatry.

[16] Mithoefer M.C., Feduccia A.A., Jerome L., Mithoefer A., Wagner M., Walsh Z., Hamilton S., Yazar-Klosinski B., Emerson A., Doblin R. (2019). MDMA-assisted psychotherapy for treatment of PTSD: Study design and rationale for phase 3 trials based on pooled analysis of six phase 2 randomized controlled trials. Psychopharmacology.

[17] United Nations Office on Drugs and Crime. www.unodc.org/documents/scientific/Global_SMART_Update_11_web.pdf.

[18] Brunt T.M., Poortman A., Niesink R.J.M., Van Den Brink W. (2011). Instability of the ecstasy market and a new kid on the block: Mephedrone. J. Psychopharmacol..

[19] Ramsey J., Dargan P.I., Smyllie M., Davies S., Button J., Holt D.W., Wood D.M. (2010). Buying “legal” recreational drugs does not mean that you are not breaking the law. QJM Int. J. Med..

[20] European Monitoring Centre for Drugs and Drug Addiction. https://www.emcdda.europa.eu/system/files/publications/13236/TDAT20001ENN_web.pdf.

[21] Guirguis A. (2017). New psychoactive substances: A public health issue. Int. J. Pharm. Pract..

[22] Gonçalves J.L., Alves V.L., Aguiar J., Teixeira H.M., Câmara J.S. (2019). Synthetic cathinones: An evolving class of new psychoactive substances. Crit. Rev. Toxicol..

[23] THC Pharm GmbH. http://usualredant.de/downloads/analyse-thc-pharm-spice-jwh-018.pdf.

[24] Gunderson E.W., Haughey H.M., Ait-Daoud N., Joshi A.S., Hart C.L. (2012). “Spice” and “K2” Herbal Highs: A Case Series and Systematic Review of the Clinical Effects and Biopsychosocial Implications of Synthetic Cannabinoid Use in Humans. Am. J. Addict..

[25] Simolka K., Lindigkeit R., Schiebel H.-M., Papke U., Ernst L., Beuerle T. (2012). Analysis of synthetic cannabinoids in “spice-like” herbal highs: Snapshot of the German market in summer 2011. Anal. Bioanal. Chem..

[26] McCarthy L.E., Borison H.L. (1981). Antiemetic Activity of N-Methyllevonantradol and Nabilone in Cisplatin-Treated Cats. J. Clin. Pharmacol..

[27] Barbado M.V., Medrano M., Caballero-Velázquez T., Álvarez-Laderas I., Sánchez-Abarca L.I., García-Guerrero E., Martín-Sánchez J., Rosado I.V., Piruat J.I., Gonzalez-Naranjo P. (2017). Cannabinoid derivatives exert a potent anti-myeloma activity both in vitro and in vivo. Int. J. Cancer.

[28] Del Rio M.J., Velez-Pardo C. (2008). Paraquat induces apoptosis in human lymphocytes: Protective and rescue effects of glucose, cannabinoids and insulin-like growth factor-1. Growth Factors.

[29] Fichna J., Bawa M., Thakur G.A., Tichkule R., Makriyannis A., McCafferty D.-M., Sharkey K.A., Storr M. (2014). Cannabinoids Alleviate Experimentally Induced Intestinal Inflammation by Acting at Central and Peripheral Receptors. PLoS ONE.

[30] Gurley S.N., Abidi A.H., Allison P., Guan P., Duntsch C., Robertson J.H., Kosanke S.D., Keir S.T., Bigner D.D., Elberger A.J. (2012). Mechanism of anti-glioma activity and in vivo efficacy of the cannabinoid ligand KM-233. J. Neurooncol..

[31] Howlett A.C. (2002). International Union of Pharmacology. XXVII. Classification of Cannabinoid Receptors. Pharmacol. Rev..

[32] Mbvundula E.C., Bunning R.A.D., Rainsford K.D. (2005). Effects of cannabinoids on nitric oxide production by chondrocytes and proteoglycan degradation in cartilage. Biochem. Pharmacol..

[33] Tolón R.M., Núñez E., Pazos M.R., Benito C., Castillo A.I., Martínez-Orgado J.A., Romero J. (2009). The activation of cannabinoid CB2 receptors stimulates in situ and in vitro beta-amyloid removal by human macrophages. Brain Res..

[34] Velez-Pardo C., Jimenez-Del-Rio M., Lores-Arnaiz S., Bustamante J. (2010). Protective Effects of the Synthetic Cannabinoids CP55,940 and JWH-015 on Rat Brain Mitochondria upon Paraquat Exposure. Neurochem. Res..

[35] Urits I., Borchart M., Hasegawa M., Kochanski J., Orhurhu V., Viswanath O. (2019). An Update of Current Cannabis-Based Pharmaceuticals in Pain Medicine. Pain Ther..

[36] European Monitoring Centre for Drugs and Drug Addiction. https://www.emcdda.europa.eu/system/files/publications/13838/TDAT21001ENN.pdf.

[37] Scourfield A., Flick C., Ross J., Wood D.M., Thurtle N., Stellmach D., Dargan P.I. (2019). Synthetic cannabinoid availability on darknet drug markets—Changes during 2016–2017. Toxicol. Commun..

[38] Le Boisselier R., Alexandre J., Lelong-Boulouard V., Debruyne D. (2017). Focus on cannabinoids and synthetic cannabinoids. Clin. Pharmacol. Ther..

[39] Brunt T.M., Atkinson A.M., Nefau T., Martinez M., Lahaie E., Malzcewski A., Pazitny M., Belackova V., Brandt S.D. (2017). Online test purchased new psychoactive substances in 5 different European countries: A snapshot study of chemical composition and price. Int. J. Drug Policy.

[40] Johanson C.E., Uhlenhuth E.H. (1981). Drug preference and mood in humans: Repeated assessment of d-amphetamine. Pharmacol. Biochem. Behav..

[41] Valente M.J., de Pinho P.G., de Lourdes Bastos M., Carvalho F., Carvalho M. (2014). Khat and synthetic cathinones: A review. Arch. Toxicol..

[42] United Nations Office on Drugs and Crime. https://www.unodc.org/documents/scientific/NPS_Report.pdf.

[43] German C.L., Fleckenstein A.E., Hanson G.R. (2014). Bath salts and synthetic cathinones: An emerging designer drug phenomenon. Life Sci..

[44] National Institute on Drug Abuse. https://www.drugabuse.gov/publications/drugfacts/synthetic-cathinones-bath-salts.

[45] Pieprzyca E., Skowronek R., Nižnanský Ľ., Czekaj P. (2020). Synthetic cathinones—From natural plant stimulant to new drug of abuse. Eur. J. Pharmacol..

[46] Calinski D.M., Kisor D.F., Sprague J.E. (2019). A review of the influence of functional group modifications to the core scaffold of synthetic cathinones on drug pharmacokinetics. Psychopharmacology.

[47] Alcohol and Drug Foundation. https://adf.org.au/drug-facts/synthetic-cathinones/.

[48] Marusich J.A., Lefever T.W., Blough B.E., Thomas B.F., Wiley J.L. (2016). Pharmacological effects of methamphetamine and alpha-PVP vapor and injection. Neurotoxicology.

[49] Taschwer M., Weiß J.A., Kunert O., Schmid M.G. (2014). Analysis and characterization of the novel psychoactive drug 4-chloromethcathinone (clephedrone). Forensic Sci. Int..

[50] European Monitoring Centre for Drugs and Drug Addiction. https://www.emcdda.europa.eu/system/files/publications/14341/EMCDDA-initial-report-3-MMC-advanced-release.pdf.

[51] Martinotti G., Santacroce R., Papanti D., Elgharably Y., Prilutskaya M., Corazza O. (2017). Synthetic Cannabinoids: Psychopharmacology, clinical aspects, and psychotic onset. CNS Neurol. Disord. -Drug Targets.

[52] Altintas M., Inanc L., Akcay Oruc G., Arpacioglu S., Gulec H. (2016). Clinical characteristics of synthetic cannabinoid-induced psychosis in relation to schizophrenia: A single-center cross-sectional analysis of concurrently hospitalized patients. Neuropsychiatr. Dis. Treat..

[53] Gurney S.M.R., Scott K.S., Kacinko S.L., Presley B.C., Logan B.K., Gurney S.M.R., Scott K.S., Kacinko S.L., Presley B.C., Logan B.K. (2014). Pharmacology, toxicology, and adverse effects of synthetic cannabinoid drugs. Forensic Sci. Rev..

[54] Radhakrishnan R., Wilkinson S.T., D’Souza D.C. (2014). Gone to Pot—A review of the association between cannabis and psychosis. Front. Psychiatry.

[55] Spaderna M., Addy P.H., D’Souza D.C. (2013). Spicing things up: Synthetic cannabinoids. Psychopharmacology.

[56] Winstock A.R., Barratt M.J. (2013). Synthetic cannabis: A comparison of patterns of use and effect profile with natural cannabis in a large global sample. Drug Alcohol Depend..

[57] Pantano F., Graziano S., Pacifici R., Busardò F.P., Pichini S. (2019). New Psychoactive Substances: A Matter of Time. Curr. Neuropharmacol..

[58] Badowski M., Yanful P.K. (2018). Dronabinol oral solution in the management of anorexia and weight loss in AIDS and cancer. Ther. Clin. Risk Manag..

[59] Schimrigk S., Marziniak M., Neubauer C., Kugler E.M., Werner G., Abramov-Sommariva D. (2017). Dronabinol Is a Safe Long-Term Treatment Option for Neuropathic Pain Patients. Eur. Neurol..

[60] Tsang C.C., Giudice M.G. (2016). Nabilone for the Management of Pain. Pharmacother. J. Hum. Pharmacol. Drug Ther..

[61] United Nations Office on Drugs and Crime. https://www.unodc.org/documents/scientific/STNAR48_Rev.1_ebook.pdf.

[62] United Nations Office on Drugs and Crime. https://www.unodc.org/documents/scientific/Synthetic_Cannabinoids.pdf.

[63] Matsuda L.A., Lolait S.J., Brownstein M.J., Young A.C., Bonner T.I. (1990). Structure of a cannabinoid receptor and functional expression of the cloned cDNA. Nature.

[64] Mackie K. (2008). Cannabinoid Receptors: Where They are and What They do. J. Neuroendocrinol..

[65] European Monitoring Centre for Drugs and Drug Addiction. https://www.emcdda.europa.eu/system/files/publications/537/Spice-Thematic-paper-final-version.pdf.

[66] Karila L., Benyamina A., Blecha L., Cottencin O., Billieux J. (2017). The Synthetic Cannabinoids Phenomenon. Curr. Pharm. Des..

[67] Lindigkeit R., Boehme A., Eiserloh I., Luebbecke M., Wiggermann M., Ernst L., Beuerle T. (2009). Spice: A never ending story?. Forensic Sci. Int..

[68] Simmler L., Buser T., Donzelli M., Schramm Y., Dieu L.-H., Huwyler J., Chaboz S., Hoener M., Liechti M. (2013). Pharmacological characterization of designer cathinones in vitro. Br. J. Pharmacol..

[69] Docherty J.R., Alsufyani H.A. (2021). Pharmacology of Drugs Used as Stimulants. J. Clin. Pharmacol..

[70] Karila L., Megarbane B., Cottencin O., Lejoyeux M. (2014). Synthetic Cathinones: A New Public Health Problem. Curr. Neuropharmacol..

[71] Cameron K., Kolanos R., Verkariya R., De Felice L., Glennon R.A. (2013). Mephedrone and methylenedioxypyrovalerone (MDPV), major constituents of “bath salts,” produce opposite effects at the human dopamine transporter. Psychopharmacology.

[72] Green A.R., King M.V., Shortall S.E., Fone K.C.F. (2014). The preclinical pharmacology of mephedrone; not just MDMA by another name. Br. J. Pharmacol..

[73] Prosser J.M., Nelson L.S. (2012). The Toxicology of Bath Salts: A Review of Synthetic Cathinones. J. Med. Toxicol..

[74] Goldberg J., Gardos G., Cole J.O. (1973). A Controlled Evaluation of Pyrovalerone in Chronically Fatigued Volunteers. Int. Pharmacopsychiatry.

[75] Mariani J.J., Khantzian E.J., Levin F.R. (2014). The self-medication hypothesis and psychostimulant treatment of cocaine dependence: An update. Am. J. Addict..

[76] Global Health Data Exchange. http://ghdx.healthdata.org/gbd-results-tool?params=gbd-api-2019-permalink/d780dffbe8a381b25e1416884959e88b.

[77] World Health Organization. https://apps.who.int/iris/bitstream/handle/10665/254610/WHO-MSD-MER-2017.2-eng.pdf.

[78] World Health Organization. https://www.who.int/news-room/fact-sheets/detail/depression.

[79] Curzon G. (1988). Serotonergic mechanisms of depression. Clin. Neuropharmacol..

[80] American Psychological Association. https://www.apa.org/depression-guideline/guideline.pdf.

[81] Katz D.P., Bhattacharya D., Bhattacharya S., Deruiter J., Clark C.R., Suppiramaniam V., Dhanasekaran M. (2014). Synthetic cathinones: “A khat and mouse game”. Toxicol. Lett..

[82] National Center for Biotechnology Information. https://www.ncbi.nlm.nih.gov/books/NBK557676/.

[83] World Health Organization. https://www.who.int/news-room/fact-sheets/detail/obesity-and-overweight.

[84] Hu F.B. (2008). Obesity Epidemiology.

[85] Hruby A., Hu F.B. (2015). The Epidemiology of Obesity: A Big Picture. Pharmacoeconomics.

[86] Kelly T., Yang W., Chen C.-S., Reynolds K., He J. (2008). Global burden of obesity in 2005 and projections to 2030. Int. J. Obes..

[87] Canning H., Goff D., Leach M.J., Miller A.A., Tateson J.E., Wheatley P.L. (1979). The involvement of dopamine in the central actions of bupropion, a new antidepressant [proceedings]. Br. J. Pharmacol..

[88] Markantonis S.L., Kyroudis A., Beckett A.H. (1986). The stereoselective metabolism of dimethylpropion and monomethylpropion. Biochem. Pharmacol..

[89] Seaton D.A., Duncan L.J.P., Rose K., Scott A.M. (1961). Diethylpropion in the Treatment of “Refractory” Obesity. BMJ.

[90] Wilens T.E., Spencer T.J. (2010). Understanding Attention-Deficit/Hyperactivity Disorder from Childhood to Adulthood. Postgrad. Med..

[91] American Psychiatric Association. https://www.psychiatry.org/patients-families/adhd/what-is-adhd.

[92] Danielson M.L., Bitsko R.H., Ghandour R.M., Holbrook J.R., Kogan M.D., Blumberg S.J. (2018). Prevalence of Parent-Reported ADHD Diagnosis and Associated Treatment among U.S. Children and Adolescents, 2016. J. Clin. Child Adolesc. Psychol..

[93] Islam F.A., Choundry Z., Duffy W. (2015). What to do when adolescents with ADHD self-medicate with bath salts. Curr. Psychiatry.

[94] Chen L., Deng H., Cui H., Fang J., Zuo Z., Deng J., Li Y., Wang X., Zhao L. (2018). Inflammatory responses and inflammation-associated diseases in organs. Oncotarget.

[95] Selvi E., Lorenzini S., Garcia-Gonzalez E., Maggio R., Lazzerini P.E., Capecchi P.L., Balistreri E., Spreafico A., Niccolini S., Pompella G. (2008). Inhibitory effect of synthetic cannabinoids on cytokine production in rheumatoid fibroblast-like synoviocytes. Clin. Exp. Rheumatol..

[96] Idris A.I., van ’t Hof R.J., Greig I.R., Ridge S.A., Baker D., Ross R.A., Ralston S.H. (2005). Regulation of bone mass, bone loss and osteoclast activity by cannabinoid receptors. Nat. Med..

[97] Gui H., Liu X., Liu L., Su D., Dai S. (2015). Activation of cannabinoid receptor 2 attenuates synovitis and joint distruction in collagen-induced arthritis. Immunobiology.

[98] Scott D.L., Wolfe F., Huizinga T.W.J. (2010). Rheumatoid arthritis. Lancet.

[99] Harth M., Nielson W.R. (2019). Pain and affective distress in arthritis: Relationship to immunity and inflammation. Expert Rev. Clin. Immunol..

[100] National Center for Biotechnology Information. https://www.ncbi.nlm.nih.gov/books/NBK507704/.

[101] Hong J.-I., Park I.Y., Kim H.A. (2020). Understanding the Molecular Mechanisms Underlying the Pathogenesis of Arthritis Pain Using Animal Models. Int. J. Mol. Sci..

[102] Tateiwa D., Yoshikawa H., Kaito T. (2019). Cartilage and Bone Destruction in Arthritis: Pathogenesis and Treatment Strategy: A Literature Review. Cells.

[103] Azizieh F.Y., Al Jarallah K., Shehab D., Gupta R., Dingle K., Raghupathy R. (2017). Patterns of circulatory and peripheral blood mononuclear cytokines in rheumatoid arthritis. Rheumatol. Int..

[104] Brennan F.M., Mcinnes I.B. (2008). Evidence that cytokines play a role in rheumatoid arthritis. J. Clin. Investig..

[105] Oliviero F., Bindoli S., Scanu A., Feist E., Doria A., Galozzi P., Sfriso P. (2020). Autoinflammatory Mechanisms in Crystal-Induced Arthritis. Front. Med..

[106] Zamora R., Vodovotz Y., Billiar T.R. (2000). Inducible Nitric Oxide Synthase and Inflammatory Diseases. Mol. Med..

[107] Abramson S.B. (2008). Nitric oxide in inflammation and pain associated with osteoarthritis. Arthritis Res. Ther..

[108] Goggs R., Carter S.D., Schulze-Tanzil G., Shakibaei M., Mobasheri A. (2003). Apoptosis and the loss of chondrocyte survival signals contribute to articular cartilage degradation in osteoarthritis. Vet. J..

[109] Hwang H., Kim H. (2015). Chondrocyte Apoptosis in the Pathogenesis of Osteoarthritis. Int. J. Mol. Sci..

[110] Stefanovic-Racic M., Taskiran D., Georgescu H.I., Evans C.H. (1995). Modulation of chondrocyte proteoglycan synthesis by endogeneously produced nitric oxide. Inflamm Res..

[111] Cabral G.A., Rogers T.J., Lichtman A.H. (2015). Turning Over a New Leaf: Cannabinoid and Endocannabinoid Modulation of Immune Function. J. Neuroimmune Pharmacol..

[112] Ungaro R., Mehandru S., Allen P.B., Peyrin-Biroulet L., Colombel J.-F. (2017). Ulcerative colitis. Lancet.

[113] National Center for Biotechnology Information. https://www.ncbi.nlm.nih.gov/books/NBK459282/.

[114] Collins P., Rhodes J. (2006). Ulcerative colitis: Diagnosis and management. BMJ.

[115] Halme L. (2006). Family and twin studies in inflammatory bowel disease. World J. Gastroenterol..

[116] Moller F.T., Andersen V., Wohlfahrt J., Jess T. (2015). Familial Risk of Inflammatory Bowel Disease: A Population-Based Cohort Study 1977–2011. Am. J. Gastroenterol..

[117] Ananthakrishnan A.N., Higuchi L.M., Huang E.S., Khalili H., Rischter J.M., Fuchs C.S., Chan A.T. (2012). Aspirin, Nonsteroidal Anti-inflammatory Drug Use, and Risk for Crohn Disease Ulcerative Colitis: A Cohort Study. Ann. Intern. Med..

[118] Engel M.A., Neurath M.F. (2010). New pathophysiological insights and modern treatment of IBD. J. Gastroenterol..

[119] Terry R., Chintanaboina J., Patel D., Lippert B., Haner M., Price K., Tracy A., Lalos A., Wakeley M., Gutierrez L.S. (2019). Expression of WIF-1 in inflammatory bowel disease. Histol. Histopathol..

[120] Yamamoto-Furusho J.K., Fonseca-Camarillo G., Furuzawa-Carballeda J., Sarmiento-Aguilar A., Barreto-Zuñiga R., Martínez-Benitez B., Lara-Velazquez M.A. (2018). Caspase recruitment domain (CARD) family (CARD9, CARD10, CARD11, CARD14 and CARD15) are increased during active inflammation in patients with inflammatory bowel disease. J. Inflamm..

[121] Wéra O., Lancellotti P., Oury C. (2016). The Dual Role of Neutrophils in Inflammatory Bowel Diseases. J. Clin. Med..

[122] Rosales C. (2018). Neutrophil: A Cell with Many Roles in Inflammation or Several Cell Types?. Front. Physiol..

[123] Fournier B.M., Parkos C.A. (2012). The role of neutrophils during intestinal inflammation. Mucosal Immunol..

[124] Papayannopoulos V., Zychlinsky A. (2009). NETs: A new strategy for using old weapons. Trends Immunol..

[125] Tecchio C., Cassatella M.A. (2016). Neutrophil-derived chemokines on the road to immunity. Semin. Immunol..

[126] Stevenson N.J., Haan S., McClurg A.E., McGrattan M.J., Armstrong M.A., Heinrich P.C., Johnston J.A. (2004). The Chemoattractants, IL-8 and Formyl-Methionyl-Leucyl-Phenylalanine, Regulate Granulocyte Colony-Stimulating Factor Signaling by Inducing Suppressor of Cytokine Signaling-1 Expression. J. Immunol..

[127] Ferreira C., Almeida C., Tenreiro S., Quintas A. (2020). Neuroprotection or Neurotoxicity of Illicit Drugs on Parkinson’s Disease. Life.

[128] Bose S., Cho J. (2013). Role of chemokine CCL2 and its receptor CCR2 in neurodegenerative diseases. Arch. Pharm. Res..

[129] National Center for Biotechnology Information. https://www.statpearls.com/ArticleLibrary/viewarticle/26674.

[130] DeMaagd G., Philip A. (2015). Parkinson’s Disease and Its Management: Part 1: Disease Entity, Risk Factors, Pathophysiology, Clinical Presentation, and Diagnosis. Pharm. Ther..

[131] Lees A.J., Hardy J., Revesz T. (2009). Parkinson’s disease. Lancet.

[132] Cabreira V., Massano J. (2019). Doença de Parkinson: Revisão Clínica e Atualização. Acta Med. Port..

[133] Jankovic J., Tan E.K. (2020). Parkinson’s disease: Etiopathogenesis and treatment. J. Neurol. Neurosurg. Psychiatry.

[134] Ascherio A., Schwarzschild M.A. (2016). The epidemiology of Parkinson’s disease: Risk factors and prevention. Lancet Neurol..

[135] Klein C., Westenberger A. (2012). Genetics of Parkinson’s Disease. Cold Spring Harb. Perspect. Med..

[136] Recasens A., Dehay B. (2014). Alpha-synuclein spreading in Parkinson’s disease. Front. Neuroanat..

[137] Percário S., da Silva Barbosa A., Varela E.L.P., Gomes A.R.Q., Ferreira M.E.S., de Nazaré Araújo Moreira T., Dolabela M.F. (2020). Oxidative Stress in Parkinson’s Disease: Potential Benefits of Antioxidant Supplementation. Oxid. Med. Cell. Longev..

[138] Ryan B.J., Hoek S., Fon E.A., Wade-Martins R. (2015). Mitochondrial dysfunction and mitophagy in Parkinson’s: From familial to sporadic disease. Trends Biochem. Sci..

[139] Guo J., Zhao X., Li Y., Li G., Liu X. (2018). Damage to dopaminergic neurons by oxidative stress in Parkinson’s disease (Review). Int. J. Mol. Med..

[140] You L.-H., Li F., Wang L., Zhao S.-E., Wang S.-M., Zhang L.-L., Zhang L.-H., Duan X.-L., Yu P., Chang Y.-Z. (2015). Brain iron accumulation exacerbates the pathogenesis of MPTP-induced Parkinson’s disease. Neuroscience.

[141] Li L., Zhang C., Chen G.Y.J., Zhu B., Chai C., Xu Q., Tan E., Zhu Q., Lim K., Yao S.Q. (2014). A sensitive two-photon probe to selectively detect monoamine oxidase B activity in Parkinson’s disease models. Nat. Commun..

[142] Scudamore O., Ciossek T. (2018). Increased Oxidative Stress Exacerbates α-Synuclein Aggregation In Vivo. J. Neuropathol. Exp. Neurol..

[143] Musgrove R.E., Helwig M., Bae E., Aboutalebi H., Lee S., Ulusoy A., Di Monte D.A. (2019). Oxidative stress in vagal neurons promotes parkinsonian pathology and intercellular α-synuclein transfer. J. Clin. Investig..

[144] Berry C., La Vecchia C., Nicotera P. (2010). Paraquat and Parkinson’s disease. Cell Death Differ..

[145] Schachter A.S., Davis K.L. (2000). Alzheimer’s disease. Dialogues Clin. Neurosci..

[146] Shinohara M., Sato N., Shimamura M., Kurinami H., Hamasaki T., Chatterjee A., Rakugi H., Morishita R. (2014). Possible modification of Alzheimer’s disease by statins in midlife: Interactions with genetic and non-genetic risk factors. Front. Aging Neurosci..

[147] Weller J., Budson A. (2018). Current understanding of Alzheimer’s disease diagnosis and treatment. F1000Research.

[148] National Center for Biotechnology Information. https://www.statpearls.com/ArticleLibrary/viewarticle/17423.

[149] Silva M.V.F., de Loures C.M.G., Alves L.C.V., de Souza L.C., Borges K.B.G., das Carvalho M.G. (2019). Alzheimer’s disease: Risk factors and potentially protective measures. J. Biomed. Sci..

[150] Giri M., Lü Y., Zhang M. (2016). Genes associated with Alzheimer’s disease: An overview and current status. Clin. Interv. Aging.

[151] Lu F.-P., Lin K.-P., Kuo H.-K. (2009). Diabetes and the Risk of Multi-System Aging Phenotypes: A Systematic Review and Meta-Analysis. PLoS ONE.

[152] Zhao X.-S., Peng J., Wu Q., Ren Z., Pan L.-H., Tang Z.-H., Jiang Z.-S., Wang G.-X., Liu L.-S. (2016). Imbalanced cholesterol metabolism in Alzheimer’s disease. Clin. Chim. Acta.

[153] Karran E., Mercken M., De Strooper B. (2011). The amyloid cascade hypothesis for Alzheimer’s disease: An appraisal for the development of therapeutics. Nat. Rev. Drug Discov..

[154] Hardy J., Selkoe D.J. (2002). The Amyloid Hypothesis of Alzheimer’s Disease: Progress and Problems on the Road to Therapeutics. Science.

[155] Murphy M.P., LeVine H. (2010). Alzheimer’s Disease and the Amyloid-β Peptide. J. Alzheimer’s Dis..

[156] Zhang Y.W., Thompson R., Zhang H., Xu H. (2011). APP processing in Alzheimer’s disease. Mol. Brain.

[157] García-González L., Pilat D., Baranger K., Rivera S. (2019). Emerging Alternative Proteinases in APP Metabolism and Alzheimer’s Disease Pathogenesis: A Focus on MT1-MMP and MT5-MMP. Front. Aging Neurosci..

[158] Blurton-Jones M., LaFerla F. (2006). Pathways by Which A? Facilitates Tau Pathology. Curr. Alzheimer Res..

[159] Rajmohan R., Reddy P.H. (2017). Amyloid-Beta and Phosphorylated Tau Accumulations Cause Abnormalities at Synapses of Alzheimer’s disease Neurons. J. Alzheimer’s Dis..

[160] Simon V., Ho D.D., Abdool Karim Q. (2006). HIV/AIDS epidemiology, pathogenesis, prevention, and treatment. Lancet.

[161] Boasso A., Shearer G.M., Chougnet C. (2009). Immune dysregulation in human immunodeficiency virus infection: Know it, fix it, prevent it?. J. Intern. Med..

[162] Schwetz T.A., Fauci A.S. (2018). The Extended Impact of Human Immunodeficiency Virus/AIDS Research. J. Infect. Dis..

[163] Boissé L., Gill M.J., Power C. (2008). HIV Infection of the Central Nervous System: Clinical Features and Neuropathogenesis. Neurol. Clin..

[164] Heaton R.K., Franklin D.R., Ellis R.J., McCutchan J.A., Letendre S.L., LeBlanc S., Corkran S.H., Duarte N.A., Clifford D.B., Woods S.P. (2011). HIV-associated neurocognitive disorders before and during the era of combination antiretroviral therapy: Differences in rates, nature, and predictors. J. Neurovirol..

[165] Nath A., Sacktor N. (2006). Influence of highly active antiretroviral therapy on persistence of HIV in the central nervous system. Curr. Opin. Neurol..

[166] Hu S., Sheng W.S., Rock R.B. (2013). CB 2 Receptor Agonists Protect Human Dopaminergic Neurons against Damage from HIV-1 gp120. PLoS ONE.

[167] Gelman B.B., Spencer J.A., Holzer C.E., Soukup V.M. (2006). Abnormal Striatal Dopaminergic Synapses in National NeuroAIDS Tissue Consortium Subjects with HIV Encephalitis. J. Neuroimmune Pharmacol..

[168] Munson A.E., Harris L.S., Friedman M.A., Dewey W.L., Carchman R.A. (1975). Antineoplastic Activity of Cannabinoids2. JNCI J. Natl. Cancer Inst..

[169] Carracedo A., Lorente M., Egia A., Blázquez C., García S., Giroux V., Malicet C., Villuendas R., Gironella M., González-Feria L. (2006). The stress-regulated protein p8 mediates cannabinoid-induced apoptosis of tumor cells. Cancer Cell.

[170] Zhang X., Qin Y., Pan Z., Li M., Liu X., Chen X., Qu G., Zhou L., Xu M., Zheng Q. (2019). Cannabidiol Induces Cell Cycle Arrest and Cell Apoptosis in Human Gastric Cancer SGC-7901 Cells. Biomolecules.

[171] Mangal N., Erridge S., Habib N., Sadanandam A., Reebye V., Sodergren M.H. (2021). Cannabinoids in the landscape of cancer. J. Cancer Res. Clin. Oncol..

[172] Laezza C., Pagano C., Navarra G., Pastorino O., Proto M.C., Fiore D., Piscopo C., Gazzerro P., Bifulco M. (2020). The Endocannabinoid System: A Target for Cancer Treatment. Int. J. Mol. Sci..

[173] Dariš B., Verboten M.T., Knez Ž., Ferk P. (2019). Cannabinoids in cancer treatment: Therapeutic potential and legislation. Bosn. J. Basic Med. Sci..

[174] Kovalchuk O., Kovalchuk I. (2020). Cannabinoids as anticancer therapeutic agents. Cell Cycle.

[175] Notaro A., Emanuele S., Geraci F., D’Anneo A., Lauricella M., Calvaruso G., Giuliano M. (2019). WIN55,212-2-Induced Expression of Mir-29b1 Favours the Suppression of Osteosarcoma Cell Migration in a SPARC-Independent Manner. Int. J. Mol. Sci..

[176] Greish K., Mathur A., Al Zahrani R., Elkaissi S., Al Jishi M., Nazzal O., Taha S., Pittalà V., Taurin S. (2018). Synthetic cannabinoids nano-micelles for the management of triple negative breast cancer. J. Control. Release.

[177] National Center for Biotechnology Information. https://www.statpearls.com/ArticleLibrary/viewarticle/25360.

[178] Tosi P. (2013). Diagnosis and Treatment of Bone Disease in Multiple Myeloma: Spotlight on Spinal Involvement. Scientifica.

[179] Jewell S., Xiang Z., Kunthur A., Mehta P. (2015). Multiple Myeloma: Updates on Diagnosis and Management. Fed. Pract..

[180] Kyle R.A., Gertz M.A., Witzig T.E., Lust J.A., Lacy M.Q., Dispenzieri A., Fonseca R., Rajkumar S.V., Offord J.R., Larson D.R. (2003). Review of 1027 Patients with Newly Diagnosed Multiple Myeloma. Mayo Clin. Proc..

[181] Dhodapkar M.V. (2016). MGUS to myeloma: A mysterious gammopathy of underexplored significance. Blood.

[182] Cardona-Benavides I.J., de Ramón C., Gutiérrez N.C. (2021). Genetic Abnormalities in Multiple Myeloma: Prognostic and Therapeutic Implications. Cells.

[183] Mullen T.D., Obeid L.M. (2012). Ceramide and Apoptosis: Exploring the Enigmatic Connections between Sphingolipid Metabolism and Programmed Cell Death. Anticancer Agents Med. Chem..

[184] National Center for Biotechnology Information. https://www.statpearls.com/ArticleLibrary/viewarticle/56333.

[185] Zhao X., Wu Q., Gong X., Liu J., Ma Y. (2021). Osteosarcoma: A review of current and future therapeutic approaches. Biomed. Eng. Online.

[186] Arndt C.A.S., Rose P.S., Folpe A.L., Laack N.N. (2012). Common Musculoskeletal Tumors of Childhood and Adolescence. Mayo Clin. Proc..

[187] Simpson E., Brown H.L. (2018). Understanding osteosarcomas. J. Am. Acad. Physician Assist..

[188] De Gonzalez A.B., Kutsenko A., Rajaraman P. (2012). Sarcoma risk after radiation exposure. Clin. Sarcoma Res..

[189] Cancemi P., Aiello A., Accardi G., Caldarella R., Candore G., Caruso C., Ciaccio M., Cristaldi L., Di Gaudio F., Siino V. (2020). The Role of Matrix Metalloproteinases (MMP-2 and MMP-9) in Ageing and Longevity: Focus on Sicilian Long-Living Individuals (LLIs). Mediat. Inflamm..

[190] Bradshaw A.D., Sage E.H. (2001). SPARC, a matricellular protein that functions in cellular differentiation and tissue response to injury. J. Clin. Investig..

[191] National Center for Biotechnology Information. https://www.statpearls.com/ArticleLibrary/viewarticle/22272.

[192] Hanif F., Muzaffar K., Perveen K., Malhi S.M., Simjee S.U. (2017). Glioblastoma multiforme: A review of its epidemiology and pathogenesis through clinical presentation and treatment. Asian Pac. J. Cancer Prev..

[193] Sofroniew M.V., Vinters H.V. (2010). Astrocytes: Biology and pathology. Acta Neuropathol..

[194] Bush N.A.O., Chang S.M., Berger M.S. (2017). Current and future strategies for treatment of glioma. Neurosurg. Rev..

[195] Davis M. (2016). Glioblastoma: Overview of Disease and Treatment. Clin. J. Oncol. Nurs..

[196] Ellor S.V., Pagano-Young T.A., Avgeropoulos N.G. (2014). Glioblastoma: Background, Standard Treatment Paradigms, and Supportive Care Considerations. J. Law Med. Ethics.

[197] Boyle P. (2012). Triple-negative breast cancer: Epidemiological considerations and recommendations. Ann. Oncol..

[198] Mehanna J., Haddad F.G.H., Eid R., Lambertini M., Kourie H.R. (2019). Triple-negative breast cancer: Current perspective on the evolving therapeutic landscape. Int. J. Womens Health.

[199] World Cancer Research Fund. https://www.wcrf.org/dietandcancer/worldwide-cancer-data/.

[200] Dawson S.J., Provenzano E., Caldas C. (2009). Triple negative breast cancers: Clinical and prognostic implications. Eur. J. Cancer.

[201] Koo M.M., von Wagner C., Abel G.A., McPhail S., Rubin G.P., Lyratzopoulos G. (2017). Typical and atypical presenting symptoms of breast cancer and their associations with diagnostic intervals: Evidence from a national audit of cancer diagnosis. Cancer Epidemiol..

[202] Jin Q., Lu J., Wu J., Luo Y. (2017). Simultaneous removal of organic carbon and nitrogen pollutants in the Yangtze estuarine sediment: The role of heterotrophic nitrifiers. Estuar. Coast. Shelf Sci..

[203] Seong M.-W., Cho S., Noh D.-Y., Han W., Kim S.-W., Park C.-M., Park H.-W., Kim S., Kim J., Park S. (2009). Comprehensive mutational analysis of BRCA1/BRCA2 for Korean breast cancer patients: Evidence of a founder mutation. Clin. Genet..

[204] Roy R., Chun J., Powell S.N. (2012). BRCA1 and BRCA2: Different roles in a common pathway of genome protection. Nat. Rev. Cancer.

[205] Peshkin B.N., Alabek M.L., Isaacs C. (2011). BRCA1/2 mutations and triple negative breast cancers. Breast Dis..

[206] Hunter P. (2012). The inflammation theory of disease. EMBO Rep..

[207] Liang Y.-C., Huang C.-C., Hsu K.-S. (2007). The synthetic cannabinoids attenuate allodynia and hyperalgesia in a rat model of trigeminal neuropathic pain. Neuropharmacology.

[208] Bialuk I., Winnicka M.M. (2011). AM251, cannabinoids receptors ligand, improves recognition memory in rats. Pharmacol. Rep..

[209] Steffens M., Szabo B., Klar M., Rominger A., Zentner J., Feuerstein T. (2003). Modulation of electrically evoked acetylcholine release through cannabinoid cb1 receptors: Evidence for an endocannabinoid tone in the human neocortex. Neuroscience.

[210] Ryberg E., Larsson N., Sjögren S., Hjorth S., Hermansson N.-O., Leonova J., Elebring T., Nilsson K., Drmota T., Greasley P.J. (2007). The orphan receptor GPR55 is a novel cannabinoid receptor. Br. J. Pharmacol..

[211] Lichtman A.H. (2000). SR 141716A enhances spatial memory as assessed in a radial-arm maze task in rats. Eur. J. Pharmacol..

[212] Keenan C.M., Storr M.A., Thakur G.A., Wood J.T., Wager-Miller J., Straiker A., Eno M.R., Nikas S.P., Bashashati M., Hu H. (2015). AM841, a covalent cannabinoid ligand, powerfully slows gastrointestinal motility in normal and stressed mice in a peripherally restricted manner. Br. J. Pharmacol..

[213] Bort A., Alvarado-Vazquez P.A., Moracho-Vilrriales C., Virga K.G., Gumina G., Romero-Sandoval A., Asbill S. (2017). Effects of JWH015 in cytokine secretion in primary human keratinocytes and fibroblasts and its suitability for topical/transdermal delivery. Mol. Pain.

[214] Wojtowicz A.M., Oliveira S., Carlson M.W., Zawadzka A., Rousseau C.F., Baksh D. (2014). The importance of both fibroblasts and keratinocytes in a bilayered living cellular construct used in wound healing. Wound Repair Regen..

[215] Barrientos S., Stojadinovic O., Golinko M.S., Brem H., Tomic-Canic M. (2008). Growth factors and cytokines in wound healing. Wound Repair Regen..

[216] Lucattelli M., Fineschi S., Selvi E., Gonzalez E.G., Bartalesi B., De Cunto G., Lorenzini S., Galeazzi M., Lungarella G. (2016). Ajulemic acid exerts potent anti-fibrotic effect during the fibrogenic phase of bleomycin lung. Respir. Res..

[217] Hernandez-Quiles M., Broekema M.F., Kalkhoven E. (2021). PPARgamma in Metabolism, Immunity, and Cancer: Unified and Diverse Mechanisms of Action. Front. Endocrinol..

[218] Milam J.E., Keshamouni V.G., Phan S.H., Hu B., Gangireddy S.R., Hogaboam C.M., Standiford T.J., Thannickal V.J., Reddy R.C. (2008). PPAR-γ agonists inhibit profibrotic phenotypes in human lung fibroblasts and bleomycin-induced pulmonary fibrosis. Am. J. Physiol. Cell. Mol. Physiol..

[219] Liu J., Li H., Burstein S.H., Zurier R.B., Chen J.D. (2003). Activation and binding of peroxisome proliferator-activated receptor gamma by synthetic cannabinoid ajulemic acid. Mol. Pharmacol..

[220] Huizenga M.N., Wicker E., Beck V.C., Forcelli P.A. (2017). Anticonvulsant effect of cannabinoid receptor agonists in models of seizures in developing rats. Epilepsia.

[221] Griffin A., Anvar M., Hamling K., Baraban S.C. (2020). Phenotype-Based Screening of Synthetic Cannabinoids in a Dravet Syndrome Zebrafish Model. Front. Pharmacol..

[222] Bayram S., Parlar A., Arslan S.O. (2020). The curative effect of cannabinoid 2 receptor agonist on functional failure and disruptive inflammation caused by intestinal ischemia and reperfusion. Fundam. Clin. Pharmacol..

[223] Aratani Y. (2018). Myeloperoxidase: Its role for host defense, inflammation, and neutrophil function. Arch. Biochem. Biophys..

[224] Gaweł S., Wardas M., Niedworok E., Wardas P. (2004). Malondialdehyde (MDA) as a lipid peroxidation marker. Wiad. Lek..

[225] Magid L., Heymann S., Elgali M., Avram L., Cohen Y., Liraz-Zaltsman S., Mechoulam R., Shohami E. (2019). Role of CB 2 Receptor in the Recovery of Mice after Traumatic Brain Injury. J. Neurotrauma.

[226] Petzoldt A.G., Sigrist S.J. (2014). Synaptogenesis. Curr. Biol..

[227] AL-Eitan L., Alhusban A., Alahmad S. (2020). Effects of the synthetic cannabinoid XLR-11 on the viability and migration rates of human brain microvascular endothelial cells in a clinically-relevant model. Pharmacol. Rep..

[228] Otrock Z.K., Mahfouz R.A.R., Makarem J.A., Shamseddine A.I. (2007). Understanding the biology of angiogenesis: Review of the most important molecular mechanisms. Blood Cells Mol. Dis..

[229] Van Hove L., Kim K.R., Arrick D.M., Mayhan W.G. (2021). A cannabinoid type 2 (CB2) receptor agonist augments NOS-dependent responses of cerebral arterioles during type 1 diabetes. Microvasc. Res..

[230] Ramirez S.H., Hasko J., Skuba A., Fan S., Dykstra H., McCormick R., Reichenbach N., Krizbai I., Mahadevan A., Zhang M. (2012). Activation of Cannabinoid Receptor 2 Attenuates Leukocyte-Endothelial Cell Interactions and Blood-Brain Barrier Dysfunction under Inflammatory Conditions. J. Neurosci..

[231] Arrick D.M., Sun H., Patel K.P., Mayhan W.G. (2011). Chronic resveratrol treatment restores vascular responsiveness of cerebral arterioles in type 1 diabetic rats. Am. J. Physiol. Circ. Physiol..

[232] Riley A.L., Hempel B.J., Clasen M.M. (2018). Sex as a biological variable: Drug use and abuse. Physiol. Behav..

[233] Varì M.R., Pichini S., Giorgetti R., Busardò F.P. (2019). New psychoactive substances—Synthetic stimulants. WIREs Forensic Sci..

[234] Zaami S., Giorgetti R., Pichini S., Pantano F., Marinelli E., Busardò F.P. (2018). Synthetic cathinones related fatalities: An update. Eur. Rev. Med. Pharmacol. Sci..

[235] Papaseit E., Olesti E., Pérez-Mañá C., Torrens M., Fonseca F., Grifell M., Ventura M., de la Torre R., Farré M. (2021). Acute Pharmacological Effects of Oral and Intranasal Mephedrone: An Observational Study in Humans. Pharmaceuticals.

[236] Soares J., Costa V.M., Gaspar H., Santos S., de Lourdes Bastos M., Carvalho F., Capela J.P. (2019). Structure-cytotoxicity relationship profile of 13 synthetic cathinones in differentiated human SH-SY5Y neuronal cells. Neurotoxicology.

[237] Zhou X., Luethi D., Sanvee G., Bouitbir J., Liechti M., Krähenbühl S. (2019). Molecular Toxicological Mechanisms of Synthetic Cathinones on C2C12 Myoblasts. Int. J. Mol. Sci..

[238] Araújo A.M., Valente M.J., Carvalho M., da Silva D.D., Gaspar H., Carvalho F., de Lourdes Bastos M., de Pinho P.G. (2015). Raising awareness of new psychoactive substances: Chemical analysis and in vitro toxicity screening of ‘legal high’ packages containing synthetic cathinones. Arch. Toxicol..

[239] Belhadj-Tahar H., Sadeg N. (2005). Methcathinone: A new postindustrial drug. Forensic Sci. Int..

[240] Anneken J.H., Angoa-Pérez M., Kuhn D.M. (2015). 3,4-Methylenedioxypyrovalerone prevents while methylone enhances methamphetamine-induced damage to dopamine nerve endings: β-ketoamphetamine modulation of neurotoxicity by the dopamine transporter. J. Neurochem..

[241] Sewalia K., Watterson L.R., Hryciw A., Belloc A., Ortiz J.B., Olive M.F. (2018). Neurocognitive dysfunction following repeated binge-like self-administration of the synthetic cathinone 3,4-methylenedioxypyrovalerone (MDPV). Neuropharmacology.

[242] National Association of Boards of Pharmacy. https://nabp.pharmacy/news/news-releases/bath-salts-linked-to-nearly-23000-emergency-room-visits-in-2011/.

[243] Murray B.L., Murphy C.M., Beuhler M.C. (2012). Death Following Recreational Use of Designer Drug “Bath Salts” Containing 3,4-Methylenedioxypyrovalerone (MDPV). J. Med. Toxicol..

[244] Warrick B.J., Wilson J., Hedge M., Freeman S., Leonard K., Aaron C. (2012). Lethal Serotonin Syndrome after Methylone and Butylone Ingestion. J. Med. Toxicol..

[245] Mayo Clinic. https://www.mayoclinic.org/drugs-supplements/diethylpropion-oral-route/proper-use/drg-20075120.

[246] Halbsguth U., Schwanda S., Lehmann T., Ostheeren-Michaelis S., Fattinger K. (2009). Necrotising vasculitis of the skin associated with an herbal medicine containing amfepramone. Eur. J. Clin. Pharmacol..

[247] Iqbal M., Monaghan T., Redmond J. (2012). Manganese toxicity with ephedrone abuse manifesting as parkinsonism: A case report. J. Med. Case Rep..

[248] Sienkiewicz-Jarosz H. (2014). MRI brain findings in ephedrone encephalopathy associated with manganese abuse: Single-center perspective. Pol. J. Radiol..

[249] Vignali C., Moretti M., Groppi A., Osculati A.M.M., Tajana L., Morini L. (2019). Distribution of the Synthetic Cathinone α-Pyrrolidinohexiophenone in Biological Specimens. J. Anal. Toxicol..

[250] Young A.C., Schwarz E.S., Velez L.I., Gardner M. (2013). Two cases of disseminated intravascular coagulation due to “bath salts” resulting in fatalities, with laboratory confirmation. Am. J. Emerg. Med..

[251] Wyman J.F., Lavins E.S., Engelhart D., Armstrong E.J., Snell K.D., Boggs P.D., Taylor S.M., Norris R.N., Miller F.P. (2013). Postmortem Tissue Distribution of MDPV Following Lethal Intoxication by “Bath Salts”. J. Anal. Toxicol..

[252] Regunath H., Ariyamuthu V.K., Dalal P., Misra M. (2012). Bath salt intoxication causing acute kidney injury requiring hemodialysis. Hemodial. Int..

[253] Adebamiro A., Perazella M.A. (2012). Recurrent acute kidney injury following bath salts intoxication. Am. J. Kidney Dis..

[254] Schifano F., Napoletano F., Chiappini S., Guirguis A., Corkery J.M., Bonaccorso S., Ricciardi A., Scherbaum N., Vento A. (2021). New/emerging psychoactive substances and associated psychopathological consequences. Psychol. Med..

[255] Murray B., Carpenter J., Dunkley C., Moran T.P., Kiernan E.A., Rianprakaisang T., Alsukaiti W.S., Calello D.P., Kazzi Z. (2020). Single-Agent Bupropion Exposures: Clinical Characteristics and an Atypical Cause of Serotonin Toxicity. J. Med. Toxicol..

[256] Sidlak A.M., Koivisto K.O., Marino R.T., Abesamis M.G. (2020). Serotonin toxicity from isolated bupropion overdoses. Clin. Toxicol..

[257] European Monitoring Centre for Drugs and Drug Addiction. https://www.emcdda.europa.eu/attachements.cfm/att_240380_EN_TDAN15001ENN.pdf.

[258] Moss M.J., Hendrickson R.G. (2019). Serotonin Toxicity. J. Clin. Psychopharmacol..

[259] Kasick D.P., McKnight C.A., Klisovic E. (2012). “Bath salt” ingestion leading to severe intoxication delirium: Two cases and a brief review of the emergence of mephedrone use. Am. J. Drug Alcohol Abus..

[260] Coppola M., Mondola R. (2012). Synthetic cathinones: Chemistry, pharmacology and toxicology of a new class of designer drugs of abuse marketed as “bath salts” or “plant food”. Toxicol. Lett..

[261] Pedersen A.J., Reitzel L.A., Johansen S.S., Linnet K. (2013). In vitro metabolism studies on mephedrone and analysis of forensic cases. Drug Test. Anal..

[262] Adamowicz P., Tokarczyk B., Stanaszek R., Slopianka M. (2013). Fatal Mephedrone Intoxication—A Case Report. J. Anal. Toxicol..

[263] Dasgupta A. (2017). Challenges in Laboratory Detection of Unusual Substance Abuse: Issues with Magic Mushroom, Peyote Cactus, Khat, and Solvent Abuse. Adv. Clin. Chem..

[264] Pearson J.M., Hargraves T.L., Hair L.S., Massucci C.J., Clinton Frazee C., Garg U., Pietak B.R. (2012). Three Fatal Intoxications Due to Methylone. J. Anal. Toxicol..

[265] Hasegawa K., Wurita A., Minakata K., Gonmori K., Nozawa H., Yamagishi I., Watanabe K., Suzuki O. (2015). Postmortem distribution of PV9, a new cathinone derivative, in human solid tissues in a fatal poisoning case. Forensic Toxicol..

[266] Wojcieszak J., Krzemień W., Zawilska J.B. (2016). JWH-133, a Selective Cannabinoid CB2 Receptor Agonist, Exerts Toxic Effects on Neuroblastoma SH-SY5Y Cells. J. Mol. Neurosci..

[267] Almada M., Alves P., Fonseca B.M., Carvalho F., Queirós C.R., Gaspar H., Amaral C., Teixeira N.A., Correia-da-Silva G. (2020). Synthetic cannabinoids JWH-018, JWH-122, UR-144 and the phytocannabinoid THC activate apoptosis in placental cells. Toxicol. Lett..

[268] Lenzi M., Cocchi V., Cavazza L., Bilel S., Hrelia P., Marti M. (2020). Genotoxic Properties of Synthetic Cannabinoids on TK6 Human Cells by Flow Cytometry. Int. J. Mol. Sci..

[269] Sezer Y., Jannuzzi A.T., Huestis M.A., Alpertunga B. (2021). In vitro assessment of the cytotoxic, genotoxic and oxidative stress effects of the synthetic cannabinoid JWH-018 in human SH-SY5Y neuronal cells. Toxicol. Res..

[270] Couceiro J., Bandarra S., Sultan H., Bell S., Constantino S., Quintas A. (2016). Toxicological impact of JWH-018 and its phase I metabolite N-(3-hydroxypentyl) on human cell lines. Forensic Sci. Int..

[271] Ferreira C., Couceiro J., Família C., Jardim C., Antas P., Santos C.N., Outeiro T.F., Tenreiro S., Quintas A. (2019). The synthetic cannabinoid JWH-018 modulates Saccharomyces cerevisiae energetic metabolism. FEMS Yeast Res..

[272] Koller V.J., Zlabinger G.J., Auwärter V., Fuchs S., Knasmueller S. (2013). Toxicological profiles of selected synthetic cannabinoids showing high binding affinities to the cannabinoid receptor subtype CB1. Arch. Toxicol..

[273] Tomiyama K., Funada M. (2011). Cytotoxicity of synthetic cannabinoids found in “Spice” products: The role of cannabinoid receptors and the caspase cascade in the NG 108-15 cell line. Toxicol. Lett..

[274] Tomiyama K., Funada M. (2014). Cytotoxicity of synthetic cannabinoids on primary neuronal cells of the forebrain: The involvement of cannabinoid CB1 receptors and apoptotic cell death. Toxicol. Appl. Pharmacol..

[275] Cha H.J., Seong Y.-H., Song M.-J., Jeong H.-S., Shin J., Yun J., Han K., Kim Y.-H., Kang H., Kim H.S. (2015). Neurotoxicity of Synthetic Cannabinoids JWH-081 and JWH-210. Biomol. Ther..

[276] Tournebize J., Gibaja V., Kahn J.-P. (2017). Acute effects of synthetic cannabinoids: Update 2015. Subst. Abus..

[277] Giorgetti A., Busardò F.P., Tittarelli R., Auwärter V., Giorgetti R. (2020). Post-mortem toxicology: A systematic review of death cases involving synthetic cannabinoid receptor agonists. Front. Psychiatry.

[278] Tai S., Fantegrossi W.E. (2014). Synthetic Cannabinoids: Pharmacology, Behavioral Effects, and Abuse Potential. Curr. Addict. Rep..

[279] Soria M.L. (2018). Las nuevas drogas psicoactivas: Populares y peligrosas. Rev. Española Med. Leg..

[280] Tait R.J., Caldicott D., Mountain D., Hill S.L., Lenton S. (2016). A systematic review of adverse events arising from the use of synthetic cannabinoids and their associated treatment. Clin. Toxicol..

[281] Potts A.J., Cano C., Thomas S.H.L., Hill S.L. (2020). Synthetic cannabinoid receptor agonists: Classification and nomenclature. Clin. Toxicol..

[282] Winstock A., Lynskey M., Borschmann R., Waldron J. (2015). Risk of emergency medical treatment following consumption of cannabis or synthetic cannabinoids in a large global sample. J. Psychopharmacol..

[283] Zaurova M., Hoffman R.S., Vlahov D., Manini A.F. (2016). Clinical Effects of Synthetic Cannabinoid Receptor Agonists Compared with Marijuana in Emergency Department Patients with Acute Drug Overdose. J. Med. Toxicol..

